# VE-1 regulation of MAPK signaling controls sexual development in *Neurospora crassa*

**DOI:** 10.1128/mbio.02264-24

**Published:** 2024-09-16

**Authors:** Sara Cea-Sánchez, Sara Martín-Villanueva, Gabriel Gutiérrez, David Cánovas, Luis M. Corrochano

**Affiliations:** 1 Departamento de Genética, Facultad de Biología, Universidad de Sevilla, Seville, Spain; 2 Instituto de Biomedicina de Sevilla, Hospital Universitario Virgen del Rocío/CSIC/Universidad de Sevilla, Seville, Spain; Universidade de Sao Paulo, Ribeirao Preto, Sao Paulo, Brazil

**Keywords:** sexual development, MAP kinases, *Neurospora*, signal transduction, velvet genes, transcriptional regulation

## Abstract

Sexual reproduction in fungi allows genetic recombination and increases genetic diversity, allowing adaptation and survival. The velvet complex is a fungal-specific protein assembly that regulates development, pathogenesis, and secondary metabolism in response to environmental cues, such as light. In *Neurospora crassa*, this complex comprises VE-1, VE-2, and LAE-1. Deletion of *ve-1* or *ve-2*, but not *lae-1*, leads to increased conidiation (asexual spore formation) and reduced sexual development. Mutants lacking *ve-1* and/or *ve-2* are female sterile and male fertile, indicating that a VE-1/VE-2 complex regulates the development of female structures. During sexual development, we observed differential regulation of 2,117 genes in dark and 4,364 genes in light between the wild type and the ∆*ve-1* strain. The pheromone response and cell wall integrity pathways were downregulated in the ∆*ve-1* mutant, especially in light. Additionally, we found reduced levels of both total and phosphorylated MAK-1 and MAK-2 kinases. *In vitro* experiments demonstrated the binding of VE-1 and VE-2 to the promoters of *mak-1* and *mak-2*, suggesting a direct regulatory role of VE-1/VE-2 in the transcriptional control of MAPK genes to regulate sexual development. Deletion of the photosensor gene *white-collar 1* prevented the light-dependent inhibition of sexual development in the ∆*ve-1* mutant by increasing transcription of the pheromone response and cell wall integrity pathway genes to the levels in the dark. Our results support the proposal that the regulation of the MAP kinase pathways by the VE-1/VE-2 complex is a key element in transcriptional regulation that occurs during sexual development.

**IMPORTANCE:**

Sexual reproduction generates new gene combinations and novel phenotypic traits and facilitates evolution. Induction of sexual development in fungi is often regulated by environmental conditions, such as the presence of light and nutrients. The velvet protein complex coordinates internal cues and environmental signals to regulate development. We have found that VE-1, a component of the velvet complex, regulates transcription during sexual development in the fungus *Neurospora crassa*. VE-1 regulates the transcription of many genes, including those involved in mitogen-activated protein kinase (MAPK) signaling pathways that are essential in the regulation of sexual development, and regulates the activity of the MAPK pathway. Our findings provide valuable insights into how fungi respond to environmental signals and integrate them into their reproductive processes.

## INTRODUCTION

Sexual reproduction is an essential process within the life cycle of eukaryotic organisms. It serves as the primary mechanism driving genetic variation in populations and enhancing adaptability to novel environmental conditions (1, 2). Sexual reproduction has been described in numerous species throughout the fungal kingdom, emphasizing the remarkable conservation of their sexual cycle despite the diversity in their lifestyle and morphology. Indeed, sexual differentiation is a complex process that involves the formation of many distinct cell types and specialized tissues, which differ between different fungal clades (3–5).

In fungi, sexual development initiates upon reception of specific environmental signals, such as nutrients and light (6–13). The environmental signals must be transduced into the cell to promote sexual development, and it has been shown that several signal transduction pathways play key roles in fungal mating and fruiting body morphogenesis. Mitogen-activated protein (MAP) kinase cascades are conserved signaling pathways in eukaryotes that are essential for the regulation of mating and fruiting body formation in fungi (5, 14, 15). Several MAP kinase cascades have been identified in fungi: the pheromone response (PR) pathway, the cell wall integrity (CWI) pathway, and the osmosensing pathway. Blocking of the PR pathway prevents mating and fruiting body formation (14, 16–19). Additionally, other signal transduction pathways have been reported to play a role in sexual development, such as the STRIPAK pathway, the NOX pathway, and the COP-9 and PACC signal transduction pathways (20–26).

Contrary to the highly conserved MAP kinases, the velvet proteins are fungal specific. Velvet proteins are characterized by a DNA binding domain that shares structural similarity with the NF-κB mammalian transcription factors (27) and are widely distributed in fungi (28). The velvet proteins form regulatory complexes responsible for the regulation of development, secondary metabolism, and pathogenicity (20, 29–32). The observations in several fungi suggest that the velvet complex participates in the regulation of sexual development. In *Aspergillus nidulans,* two of the velvet proteins, VeA and VelB, physically interact and activate sexual development in the dark (33). In *Aspergillus flavus*, VeA, VelB, and the methyltransferase LaeA, are required for the formation of sclerotia, the structures where fruiting bodies are produced (34, 35). In *Trichoderma reesei* the VeA homolog, VEL1, also acts as a positive regulator of sexual development (36–38). In *Neurospora crassa*, the velvet complex, composed of VE-1 and VE-2, and the methyltransferase LAE-1, regulates asexual development in response to light (39, 40), and evidence for the role of the velvet complex in sexual development was previously observed as mutants in *ve-1*, *ve-2,* or *lae-1* produced less protoperithecia than the wild type (41).

*N. crassa* is a self-sterile fungus with two distinct mating types, *mat A* and *mat a*. Sexual development initiates with the formation of protoperithecia (immature female organs). Transition from protoperithecia to the perithecial stage (mature female organs) involves the formation of one or more trichogynes, specialized-receptive hyphae that extend from the protoperithecium toward male cells (macroconidia, microconidia, or hyphal fragments) of the opposite mating type by chemotropic attraction. Meiosis occurs within the fertilized perithecium while it expands and melanizes. Finally, it develops a pore (ostiole) at the tip (beak), enabling the ejection of mature ascospores, the products of meiosis, which then form a new mycelium (42, 43). The transcriptomic landscape of *N. crassa* during the formation of the fruiting body has been examined by RNA-sequencing experiments (44, 45), revealing major changes in the transcriptome of developing fruiting bodies during the sexual cycle, as well as a stage-specific expression pattern for many key genes during sexual differentiation. Similar transcriptional changes have also been found by many transcriptome analyses in other fungal species (30, 46, 47). In addition, comparative genomics and transcriptomics analysis between different fungi has allowed the identification of putative regulators of sexual development by selecting those genes that display a conserved and a stage-specific expression profile during sexual development in different lineages (3, 30, 48).

In this study, a comparative genome-wide transcriptional analysis between a wild-type strain and a deletion mutant ∆*ve-1* of *N. crassa* revealed that VE-1, together with VE-2, plays a major role as a light-dependent positive regulator of female fertility and that VE-1 controls sexual development by the regulation of components of the MAP kinase signaling transduction pathways.

## RESULTS

### Proteins VE-1, VE-2, and LAE-1 are required for female fertility during sexual development in *N. crassa*

The observations suggesting a role of the velvet complex in the formation of protoperithecia in *N. crassa* (41), and our previous results showing a link between VE-1 and blue-light photoreception in the regulation of asexual development (40) prompted us to further characterize the roles of the different components of the velvet complex in sexual development and, in particular, their connection with light.

**Fig 1:**
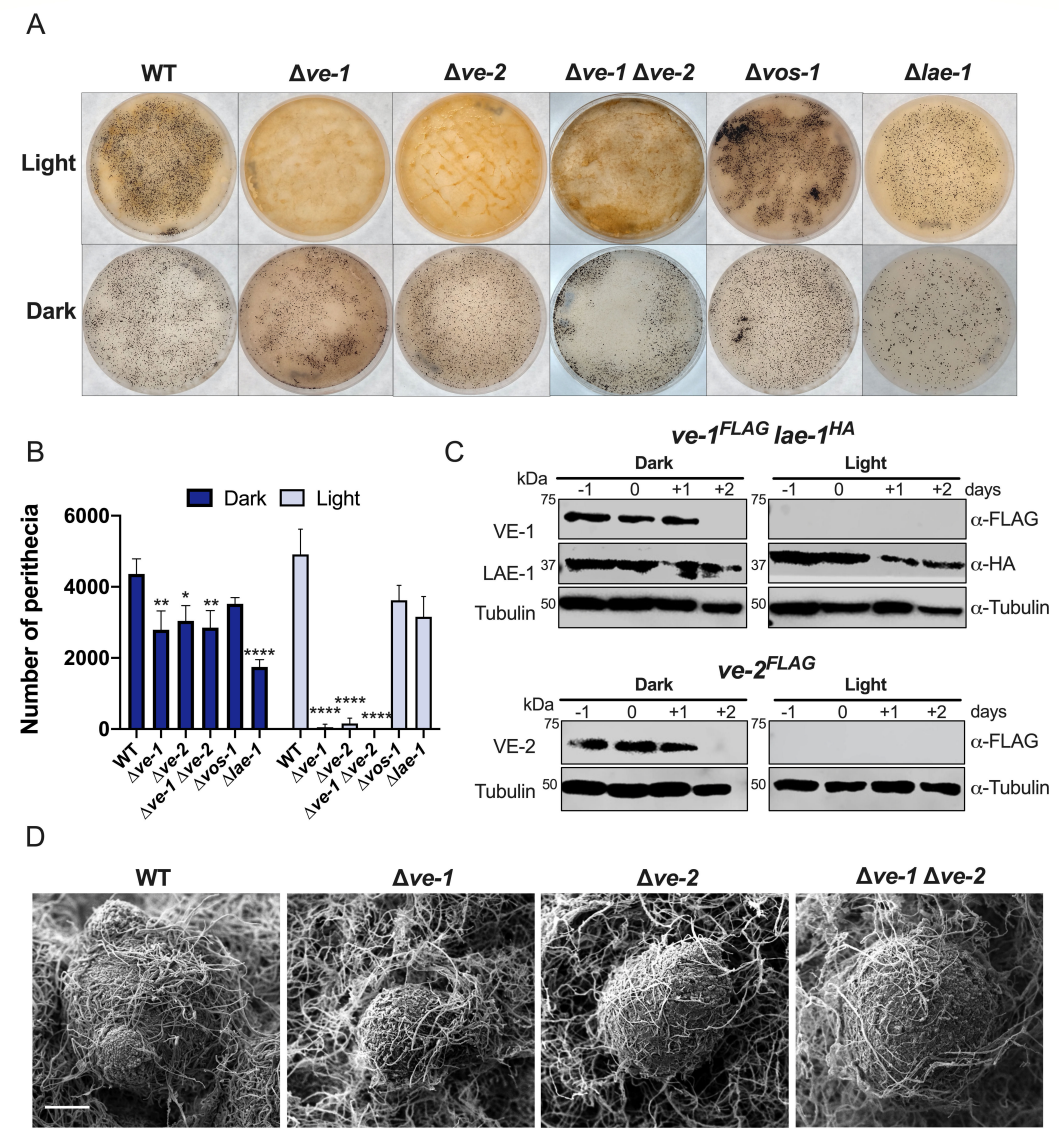
VE-1, VE-2, and LAE-1 participate in the development of female structures during sexual development. (**A**) Formation of perithecia of wild-type, ∆*ve-1,* ∆*ve-2,* ∆*ve-1* ∆*ve-2,* ∆*vos-1*, and ∆*lae-1* strains in SCM plates 7 days after fertilization in light (upper panel) and dark (lower panel). (**B**) Quantification of the total number of perithecia in plates seven days after crossing. The plot shows the mean and standard error of four independent experiments (*P < 0.05, **P < 0.01, ***P < 0.001, ****P < 0.0001). (**C**) Accumulation of proteins for the components of the velvet complex during sexual development. Protein samples from female sexual structures of strains carrying *ve-1^FLAG^*, *ve-2^FLAG^*, or *lae-1^HA^* alleles before or after fertilization at day 0 were separated by PAGE and hybridized with antibodies specific for the FLAG or HA epitopes or for tubulin. Seventy micrograms of proteins were loaded per lane. We used tubulin as a loading control. (**D**) Scanning electron microscopy (SEM) images of the perithecia of the wild-type strain and the ∆*ve-1*, ∆*ve-2*, and ∆*ve-1* ∆*ve-2* strains cultured in complete darkness for 7 days after fertilization. Scale bar = 100 µm. Samples were visualized with a JEOL 6460LV instrument.

Conidia of wild-type and mutant strains (∆*ve-1,* ∆*ve-2,* ∆*ve-1* ∆*ve-2*, ∆*vos-1*, and ∆*lae-1*) were spread on synthetic crossing medium (SCM) agar plates and incubated at 22°C in continuous darkness and continuous light. After 7 days of incubation, sexual crosses were initiated by applying a suspension of wild-type conidia of the opposite mating type, and plates were incubated for up to 7 additional days to allow perithecia maturation and ascospore formation. In the wild-type strain, initial observations within 24 h post-fertilization revealed minimal tissue differentiation, characterized by a slight increase in size and a subtle darkening in color. As expected, after 5–7 days following fertilization, a significant number of mature perithecia were observed evenly distributed over the plate (Fig. 1A). Comparisons between the wild-type strain and the ∆*ve-1* and ∆*ve-2* mutants revealed defects in sexual development in both dark and light conditions (Fig. 1A and B). The amount of perithecia in the ∆*ve-1* and ∆*ve-2* mutants was reduced to 65% when compared with the wild-type strain in the dark. The alteration of sexual development was more pronounced in light as the amount of perithecia was 2% (∆*ve-1*) or 10% (∆*ve-2*) of that observed in the wild type. The double mutant ∆*ve-1* ∆*ve-2* displayed similar alterations as each single mutant. Deletion of *ve-1* or *ve-2* also affected perithecia maturation, as shown by a reduction in the size of the perithecia and changes in the morphology of the perithecial beak (Fig. 1D; Fig. S1A and B). Ascospore release was rarely observed in crosses where ∆*ve-1* and ∆*ve-2* mutant strains acted as females. Viable ascospores were isolated after crushing the perithecia and manually collecting the ascospores. A mutation in the gene that encodes the other component of the velvet complex, *lae-1*, resulted in a less pronounced effect on perithecia formation. The ∆*lae-1* mutant only produced 40% of the number of perithecia observed in the wild-type strain in darkness and 65% of the wild-type perithecia production in light. No alterations were detected in the formation and maturation of perithecia in a mutant of another velvet gene, *vos-1* (Fig. 1A and B). The reduction in the number of perithecia was due to a reduction in the number of female structures (protoperithecia) in the ∆*ve-1* and ∆*ve-2* mutants in the early stages of sexual development before fertilization (Fig. S2), corresponding to the stages where the accumulation of the velvet complex is observed (Fig. 1C). The accumulation of VE-1 and VE-2 proteins was higher in dark than in light, whereas LAE-1 was observed at all stages of sexual development, regardless of exposure to light.

Male fertility in the mutants of the velvet complex was not affected, since conidia from all the mutant strains could fertilize protoperithecia of the wild type (Fig. S3). Our findings support the proposal that VE-1 and VE-2, and to a lesser extent LAE-1, are required for the formation of female reproductive structures during the sexual development of *N. crassa*.

### Comparative transcriptomics revealed a role of VE-1 in transcriptional regulation during sexual development

**Fig 2:**
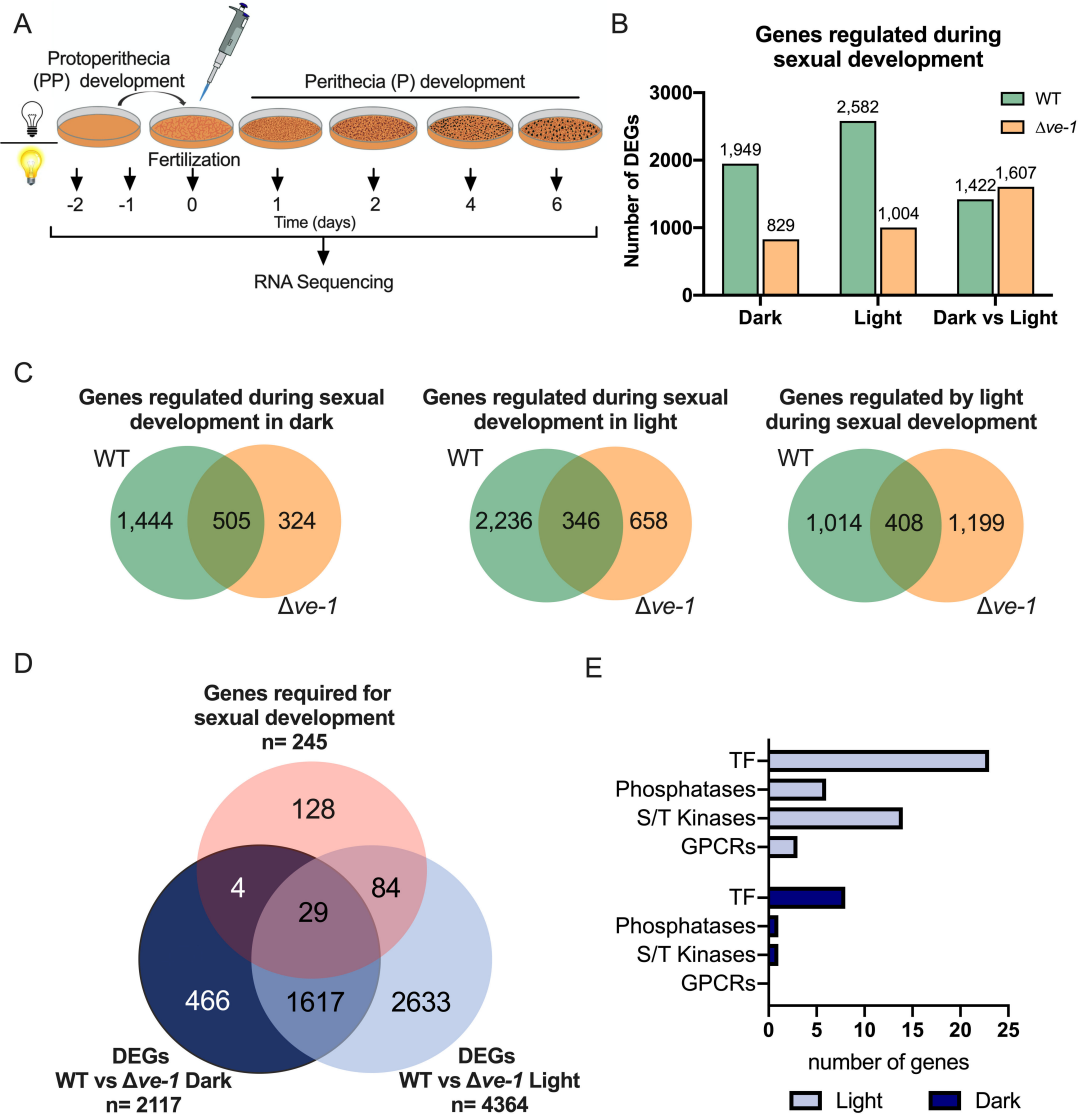
Comparison between the transcriptomes of the wild-type and the ∆*ve-1* strains during sexual development. (**A**) Schematic representation of experimental conditions for the characterization of changes in mRNA accumulation during sexual development. (**B**) Number of DEGs regulated during sexual development in the wild-type and the ∆*ve-1* strains under both experimental conditions (dark and light) and DEGs regulated by light during sexual development in each strain. DEGs were identified using maSigPro, an R package that identifies genes with differences in mRNA accumulation in time course RNAseq experiments. (**C**) Venn diagram comparing the transcriptomic responses of the wild-type and the ∆*ve-1* strains during sexual development (dark and light) and in response to light during the sexual cycle. (**D**) Venn diagram depicting genes misregulated in the ∆*ve-1* under dark and light conditions, and genes that result in a sexual phenotype when deleted as reported by Carrillo et al. (50). (**E**) Bar graph showing the number of VE-1 misregulated genes encoding G protein-coupled receptors (GPCRs), serine/threonine kinases (S/T kinases), phosphatases, and transcription factors (TF) in light and dark.

Our discovery that VE-1 was required for gene expression during conidiation, and the alterations observed in sexual development in the ∆*ve-1* mutant, prompted us to characterize the role of VE-1 in transcriptional regulation during sexual development. To identify changes in transcription due to the lack of VE-1, wild-type (FGSC#2489 mat A) and ∆*ve-1* (FGSC#11401 mat A) cultures kept in the dark or in the light from seven stages during the sexual cycle, including the last stages of protoperithecia formation (2 days and 1 day prior to fertilization) and five stages of maturation of perithecia (the fertilization day, day 0, and 1, 4, and 6 days after fertilization), were analyzed by RNA sequencing (Fig. 2A; Data set S1). Identification of differentially expressed genes (DEGs) was carried out using maSigPro, an R package that identifies genes with differences in mRNA accumulation in at least one point in time course RNAseq experiments (49).

Our results revealed a major reprogramming of the wild-type transcriptome during sexual development with 1,949 DEGs in the dark and 2,582 in the light. In contrast, a reduction in the number of DEGs during sexual development was observed in the ∆*ve-1* strain, with 829 DEGs in the dark and 1,004 DEGs in the light, corresponding to a 60% reduction under both experimental conditions (Fig. 2B; Data set S2). Furthermore, we compared the transcriptomes during the sexual development of both strains under each experimental condition (Fig. 2C). Of 1,949 DEGs that were identified in the wild-type in the dark, 74% lost their sexual-dependent regulation in the ∆*ve-1* mutant, and deletion of ∆*ve-1* led to a new set of 324 DEGs in the mutant. When cultures were exposed to light, the sexual-dependent transcriptome of wild-type and ∆*ve-1* strains barely overlapped (346 genes, approximately 15% of the wild-type transcriptome), and 658 new genes were regulated during sexual development in the ∆*ve-1* mutant. The sets of genes commonly regulated in both the wild-type and ∆*ve-1* strains were largely comprised of genes predominantly induced in the wild-type strain after fertilization, regardless of whether they were identified under dark or light conditions. Induction of those genes was severely diminished in the *ve-1* mutant (Fig. S4A and B). Principal component analysis (PCA) applied to the commonly regulated gene sets unveiled four discrete sample clusters, each aligning with the respective strain and condition. This pattern was evident solely within the DEGs associated with light conditions (Fig. S4C), but not with dark conditions (Fig. S4A and B). Altogether, these differences are consistent with a more severe phenotype of the ∆*ve-1* strain in the light than in the dark.

### VE-1 controls the expression of genes required for growth and asexual and sexual development during fruiting body formation

**Fig 3:**
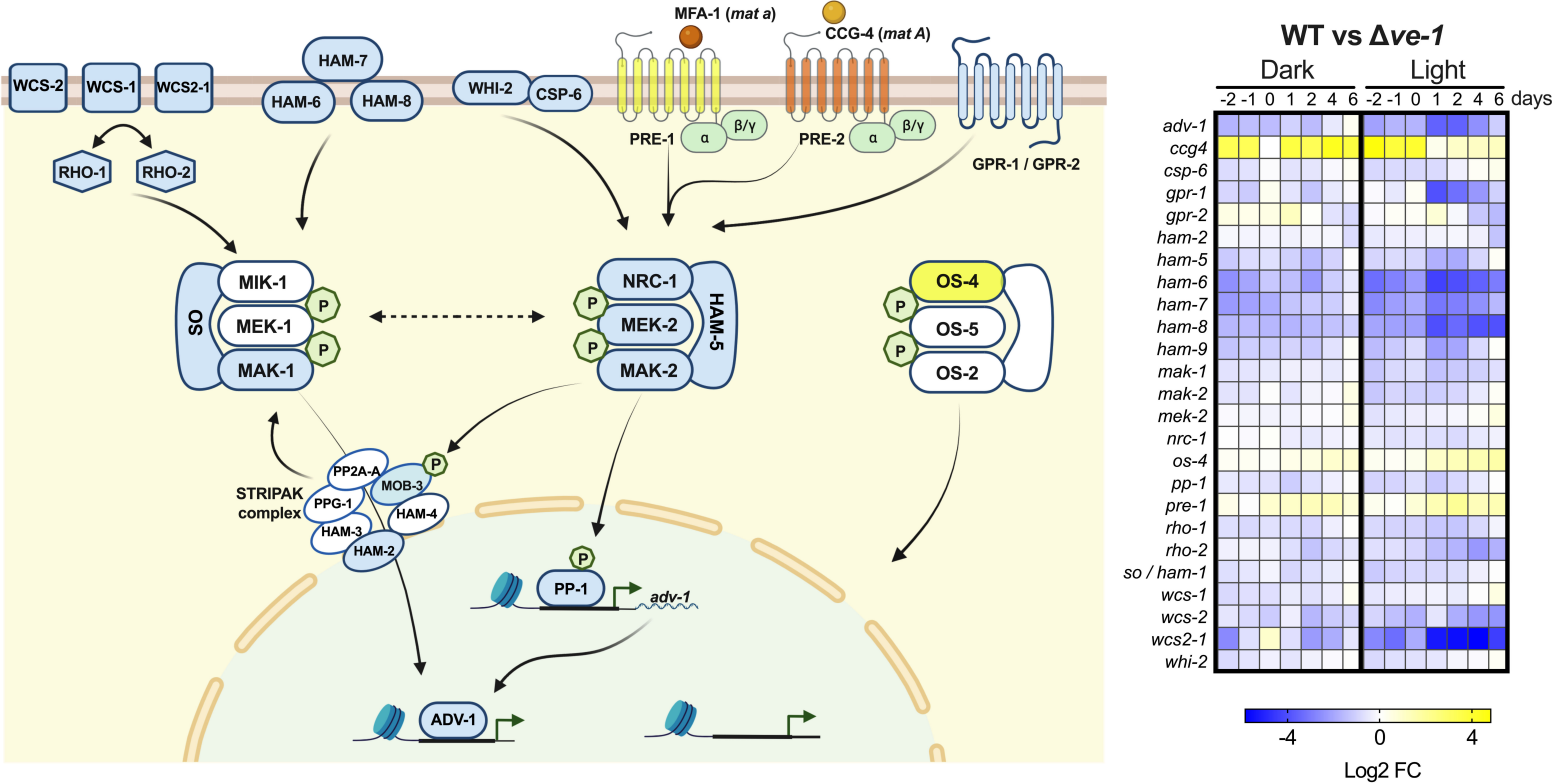
VE-1 regulates the transcription of genes in the MAP kinase signaling transduction pathways required for sexual development. (Left panel) Schematic model of components of the MAP kinase signaling pathways (from left to right, CWI, PR, and OS MAP kinase signaling cascades) that are misregulated in the ∆*ve-1 mat A* strain. Components in blue are downregulated and in yellow are upregulated in the ∆*ve-1 mat A* compared with the wild-type strain *mat A*. We included the mating type pheromone ligand MFA-1 and its cognate receptor PRE-2 specific for *mat a* mating type. MFA-1 and PRE-2 are colored in orange, as we do not have RNA expression data from *mat a* cell acting as the female partner. Green “P” indicates phosphorylation. Dotted arrow depicts crosstalk between modules of different signaling cascades. For simplicity, a number of signaling components and phosphorylation events have been left out. (Right panel) Heatmap showing differential expression values (log2 FC) of DEGs in the mutant ∆*ve-1* compared with the wild-type strain during sexual development in dark and light. The schematic model has been created with BioRender.com

To identify genes regulated by VE-1 during sexual development, we made direct comparisons of mRNA accumulation in the wild-type strain with the ∆*ve-1* strain throughout the time course of sexual development under both experimental conditions (dark and light). This analysis revealed major transcriptional changes in the ∆*ve-1* mutant when compared with the wild-type strain in dark (2,117 genes, corresponding to 21% of the *N. crassa* genome). The transcriptional effect of the *ve-1* deletion was larger in light, leading to twice as many misregulated genes (4,364 genes, about 44% of the *N. crassa* genome) (Fig. 2D; Data set S3). We noticed that a large set of genes was regulated by VE-1 in dark and light (1,646 genes, accounting for approximately 75% of DEGs in the dark). This set comprised genes that were induced after fertilization. PCA also displayed four distinct groups, but in this case, the samples of wild type in light and the ∆*ve-1* in dark overlapped, whereas ∆*ve-1* in light was clearly separated from all the others (Fig. S5), in agreement with the observed phenotypes. The set was enriched in genes involved in metabolism and transmembrane transport. An additional set of 2,717 genes was regulated by VE-1 only in light, and they included genes related to biosynthesis, reproduction and cell division, and stress response genes. To identify genes regulated by VE-1 that are essential for sexual development, we used a high-throughput phenotype characterization of the collection of *N. crassa* deletion mutants (50, 51). We identified a total of 33 VE-1-regulated genes in the dark and 113 in the light with a role in sexual development (corresponding to 46% of the *N. crassa* deletion mutants with a phenotype in sexual development) with an overlap of 29 genes identified in dark and light (Fig. 2D). Our results suggest that VE-1 plays a key role in the regulation and coordination of the transcription of genes that are required for sexual development (Fig. 3) and that this VE-1-dependent regulation is larger in light that in dark, which agrees with the more severe phenotype observed in the ∆*ve-1* mutant during sexual development in light.

Among the genes regulated by VE-1 that are required for sexual development, we identified genes for G-protein coupled receptors (GPCRs), signal transduction proteins, and transcription factors, suggesting that VE-1 regulates sexual development by altering the transcription of genes involved in signal transduction pathways (Fig. 2E and 3; Table 1; Data set S4).

**TABLE 1:** Selected DEGs with a role in sexual development

Gene ID	Name	Orthologs in *S. cerevisiae*	Sexual alterations^*a*^	FungiDB annotation	DEGs WT			DEGs WT vs ∆*ve-1*		Reference
					Dark	Light	Dark vs light	Dark	Light	
Pheromone response (PR)/female fertility and cell fusion pathway										
NCU00138	*pre-1*	*STE3*	P / A	Pheromone receptor pre-1	NO	NO	NO	NO	YES	(52)
NCU02500	*ccg4*		P / A	Clock-controlled pheromone ccg-4	YES	YES	YES	YES	NO	(53)
NCU06182	*nrc-1*	*STE11*	PP / P / A	MAPKK kinase	NO	NO	NO	NO	YES	(54)
NCU04612	*mek-2*	*STE7*	PP / P / A	Mitogen-activated protein kinase kinase 2	YES	YES	YES	NO	YES	(54)
NCU02393	*mak-2*	*FUS3/KSS1*	PP / P / A	Mitogen-activated protein kinase 2	YES	YES	YES	NO	YES	(55)
NCU00340	*pp-1*	*STE12*	PP / P / A	Transcription factor steA	YES	YES	YES	NO	YES	(55)
Cell wall integrity (CWI)										
NCU09842	*mak-1*	*MPK1*	PP / P / A	Mitogen-activated protein kinase MKC1	NO	NO	YES	NO	YES	(56)
NCU07392	*adv-1*		PP / P / A	Transcriptional regulatory protein pro-1	NO	NO	NO	NO	YES	(50, 57)
Osmosensing pathway (OS)										
NCU03071	*os-4*	*SSK22*	PP / P / A	MAP kinase kinase kinase SskB	NO	NO	NO	NO	YES	(54)
Components of CWI and cell fusion MAP kinase pathways										
NCU02794	*so/ham-1*		PP / P / A	Fso1	YES	YES	NO	NO	YES	(50, 58)
NCU03727	*ham-2*		pp / P / A	Hyphal anastamosis-2 protein	NO	NO	NO	NO	YES	(59)
NCU01789	*ham-5*		pp / P / A	Hyphal anastamosis-5 protein	NO	NO	NO	NO	YES	(60)
NCU02767	*ham-6*		PP / P / A	Hyphal anastamosis-6 protein	NO	NO	NO	YES	YES	(61)
NCU00881	*ham-7*		PP / P / A	Hyphal anastamosis-7 protein	YES	NO	NO	YES	YES	(62)
NCU02811	*ham-8*		PP / P / A	hyphal anastamosis-8 protein	NO	NO	NO	YES	YES	(61)
NCU10518	*whi-2*	*WHI2*	PP / P / A	Fso1	YES	YES	YES	YES	YES	(61, 63)
NCU08380	*csp-6*	*PSR1*	pp / P / A	Plasma membrane phosphatase required for sodium stress response	YES	YES	NO	NO	YES	(64)
NCU06205	*rco-1*	*TUP1*	PP / P / A	Transcriptional repressor rco-1	YES	NO	NO	YES	YES	(65)
Other signaling pathways										
NCU09123	*camk-1*	*CMK1/CMK2*	PP / P / A	Ca/CaM-dependent kinase-1, variant 2	YES	NO	YES	NO	YES	(66)
NCU09071	*dbf-2*	*DBF2/DBF20*	PP / P / A	AGC/NDR protein kinase	NO	NO	NO	NO	YES	(66)
NCU00786	*gpr-1*		p	G-protein coupled receptor	NO	NO	NO	NO	YES	(67)
NCU04626	*gpr-2*		p	G-protein coupled receptor	YES	YES	NO	NO	YES	(68)
NCU07399	*stk-9*	*ENV7*	PP / P / A	Serine/threonine protein kinase-9	NO	YES	YES	YES	YES	(66)
NCU04426	*div-4*	*CAK1*	pp/ p / a	Cell division protein kinase	NO	YES	NO	YES	YES	(66)
NCU03187	*nim-1*	*KIN3*	a	G2-specific protein kinase nimA	NO	NO	NO	NO	YES	(66)
NCU05658	*stk-36*		PP / P / A	Serine/threonine protein kinase-36	NO	NO	NO	NO	YES	(66)
NCU09064	*stk-53*	*KCC4/SNF1/GIN4/HSL1*	A	Serine/threonine protein kinase-53	NO	YES	NO	NO	YES	(66)
NCU00914	*stk-16*	*KIN4/FRK1*	PP / P / a	Serine/threonine protein kinase	NO	YES	YES	NO	YES	(66)
NCU00406	*vel*	*STE20/CLA4/SKM1*	a	Protein kinase CHM1	NO	YES	NO	NO	YES	(66)
NCU06685	*stk-47*	*CTK1*	PP / P / A	Protein kinase	NO	NO	NO	NO	YES	(66)
NCU06330	*dsp-3*		pp / p / a	Hypothetical protein	NO	YES	NO	YES	YES	(64)
NCU08158	*dsp-4*	*YVH1*	a	Dual specificity phosphatase	YES	YES	NO	NO	YES	(64)
NCU08948	*pph-11*	*NEM1*	pp / p / a	NIF domain-containing protein, variant 1	NO	YES	YES	NO	YES	(64)
NCU04600	*pph-8*	*PTC2/PTC3*	pp / p / a	Protein phosphatase-9	YES	YES	YES	NO	YES	(64)
NCU08301	*pp4*		pp / a	Calcineurin-like phosphoesterase	NO	YES	YES	NO	YES	(64)

^*a*^ PP, protoperithecia; P, perithecia; A, ascospores. Text in lower case indicates a reduced number of structures or abnormal structures compared with the WT. Text in upper case shows that the structure is not formed.

### VE-1 controls the expression of predicted G-protein-coupled receptor genes

Genes for 45 G-protein-coupled receptors (GPCRs) have been identified and characterized in the genome of *N. crassa* (67, 69). The absence of VE-1 led to significant differences in the accumulation of mRNAs of seven GPCR genes in the ∆*ve-1* in the dark and 21 in the light. Among them, we identified three GPCRs encoding genes known to participate in signal transduction pathways that are essential for sexual development (Fig. S6; Table 1). One of them was the *pre-1* pheromone receptor gene, which is essential for *mat A-*specific directional growth and fertility (52, 70, 71). Furthermore, we observed that other components of the PR pathway were also misregulated, such as the gene for the *mat A-*specific pheromone precursor, *ccg-4*, and the mating-type genes *mat A-1*, *mat A-2,* and *mat A-3* (Data set S3), which encode transcription factors that are required for transcriptional regulation of *ccg-4* in *mat A* cells (53).

Two other GPCR genes, *gpr-1* and *gpr-2*, play an important role in female fertility (67, 68). Protoperithecia of a ∆*gpr-1* mutant are small, weakly pigmented, and embedded in agar, and fertilized perithecia have deformed beaks without ostioles, and thus, they are incapable of ascospore ejection. Deletion of *gpr-2* provokes loss of perithecial beak phototropism resulting in beaks that bend downward (67). Those defects resemble the phenotype previously observed in the ∆*ve-1* and ∆*ve-2* strains. We observed an increase in the expression levels of *gpr-1* and *gpr-2* after fertilization in the wild-type strain in both dark and light, and this expression was downregulated in the ∆*ve-1* mutant after fertilization, when cultures were exposed to light (Fig. S6; Table 1).

### VE-1 regulates the expression of serine/threonine kinases and serine/threonine phosphatases

Serine/threonine (S/T) kinases and phosphatases play crucial roles in eukaryotic signaling pathways (5, 14, 15, 72). The genome of *N. crassa* contains 86 genes encoding serine/threonine protein kinases (50, 66, 69). Among the 77 previously characterized kinase gene mutants, 33 exhibited defects in sexual development (66). Within our set of VE-1-regulated genes, we identified 38 kinases, of which 15 were required for sexual development (Fig. S7; Table 1). These included genes encoding components of the three MAP kinase signaling pathways, specifically *nrc-1, mek-2* and *mak-2* (PR), and *mak-1* (CWI), which showed decreased mRNA levels in the ∆*ve-1* mutant, unlike *os-4* (OS), which exhibited higher mRNA levels in the ∆*ve-1* mutant. Notably more misregulated kinase genes were identified in cultures exposed to light (37 genes) compared with dark conditions (12 genes).

There are 24 serine/threonine and tyrosine protein phosphatases in the *N. crassa* genome that have been functionally characterized and are required for growth and development (50, 64). Within our list of VE-1-regulated genes, six phosphatases required during sexual development were identified (Fig. S8; Table 1).

### The expression of cell-to-cell fusion genes involved in sexual development is affected in the ∆*ve-1* mutant

Cell fusion events and cell communication play a key role in the life cycle of *N. crassa* and other filamentous fungi (73, 74). Mutants with defective hyphal fusion exhibit alterations in aerial hyphae formation, display a “flat” conidiation phenotype due to shortened aerial hyphae, are also affected in female fertility, and can not develop protoperithecia (54, 58, 59, 61, 74–76). The proteins HAM-6, HAM-7, and HAM-8 form a sensory complex on the cell wall/plasma membrane activating the CWI pathway through the kinase MAK-1 (61). In both dark and light conditions, the ∆*ve-1* mutant showed lower mRNA levels of *ham-6*, *ham-7,* and *ham-8* compared with the wild-type strain (Fig. S9; Table 1). Downregulation was also observed in the cell fusion genes *ham-9* and *whi-2*, involved in the coordination of MAK-1 and MAK-2 during cell fusion, as well as in vegetative growth and development (61, 63). Under light exposure, the ∆*ve-1* mutant exhibited lower mRNA levels in cell-to-cell fusion genes, including *so/ham-1, ham-2, ham-5,* and *mob-3*. Mutants lacking the *so/ham-1* gene (NCU02794) display a pleiotropic morphogenetic phenotype, i.e., shortened aerial hyphae, altered conidiation, and female sterility (58). HAM-5 acts as a MAP kinase scaffold during fusion, and deletion of *ham-5* results in the production of a low number of infertile protoperithecia (60). HAM-2 and MOB-3 are components of the STRIPAK complex, crucial for the nuclear accumulation of MAK-1 in a MAK-2 dependent manner through MOB-3 phosphorylation (77). Downregulation of cell fusion genes supports our observation of defects in protoperithecia formation similar that observed in cell fusion mutants (58, 61, 62).

### VE-1 controls the expression of transcription factors involved in sexual development

Regulation of gene expression by transcription factors (TFs) is crucial for proper coordination of growth and development in all organisms, and they represent the last step in signal transduction cascades. The genome of *N. crassa* harbors 312 TF genes (51, 55), among which 75 TF genes exhibited altered mRNA accumulation in the ∆*ve-1* mutant in the dark (24% of total TFs in the *N. crassa* genome). Exposure to light increased the number of misregulated TFs to 137 (46% of total TFs in *N. crassa* genome) (Fig. S10A). Most of the misregulated TF genes in the dark were also misregulated in the light (60 of 75), and approximately 60% of the genes misregulated in the light were specific for this condition. This suggests a major role for VE-1 in the regulation of TFs during sexual development in light, consistent with the more drastic phenotype of ∆*ve-1* in light. A gene ontology (GO) term analysis of the set of genes that were misregulated in each condition revealed an enrichment of TF genes involved in nitrogen metabolism, development, reproduction, growth, and cell differentiation (Fig. S10B).

Mutants in eight TFs that were misregulated in the dark and 23 in the light displayed different defects in sexual development (alterations in protoperithecia, perithecia, or ascospore formation) according to Carrillo et al. (50), and seven of them were misregulated under both conditions (Fig. S10C). Of the 22 TF genes with at least one sexual alteration in light, the mutation of 10 genes resulted in defects in two aspects of the sexual cycle, and 10 were defective in the three phenotypes tested. Thirteen genes led to alterations in protoperithecia production, with mutants in seven genes leading to a complete block in the formation of premature female organs. This group includes *fsd-1, rco-1, tcf-9, ada-3, ff-7,* and the genes that encode downstream effectors of the signal transduction pathways MAK-2/PR and MAK-1/CWI, *pp-1,* and *adv-1*, respectively (Table 1). There are several crosstalks between the MAK-1/ADV-1 and MAK-2/PP-1 pathways during the regulation of growth and development (78). For example, *pp-1* is the homolog of the *S. cerevisiae STE-12*, and in *N. crassa*, it also directly regulates the expression of *adv-1* (78, 79). The repressor RCO-1 is the homolog of *S. cerevisiae* Tup1, and it has been characterized as a pleiotropic transcription factor involved in cell fusion, growth, and development (76, 80, 81). The beak morphological phenotype of the ∆*ve-1* mutant (Fig. 1D) resembled that of the *bek-1* and *bek-2* mutants (68, 82). Accordingly, the transcription levels exhibited by both genes were lower in the absence of VE-1 (Fig. S11), although only differences in *bek-2* appeared to be statistically significant and only in light (Fig. S10). Our results suggest that VE-1 affects gene regulation during sexual development by altering mRNA accumulation of key TF genes.

### VE-1 requires the WC-1 photoreceptor for light-dependent inhibition of perithecial development

To get further insights into the role of light during sexual development, we compared the transcriptomes in light and dark for each strain to identify genes that were regulated by light during sexual development. Both strains had a similar number of genes regulated by light: 1,422 genes in the wild-type strain and 1,607 in the ∆*ve-1* strain (Fig. 2B). However, the light-dependent transcriptome during sexual development in each strain was very specific, that is, 1,014 genes only responded to light in the wild-type, 1,199 genes only responded to light in the ∆*ve-1* mutant, and only 408 genes were regulated by light in both strains (Fig. 2C). PCA of these 408 genes also revealed four discrete sample clusters, each aligning with the respective strain and condition (Fig. S4C), similar to the PCA of the commonly regulated genes (between wild type and mutant) in light (Fig. S4B). However, the set did not include a majority of genes regulated during the course of sexual development (Fig. S4C) and comprised genes known to respond to light in *N. crassa,* such as *al-2, bli-4, con* genes, *vvd , cry*, and *wc-1* (83).

**Fig 4:**
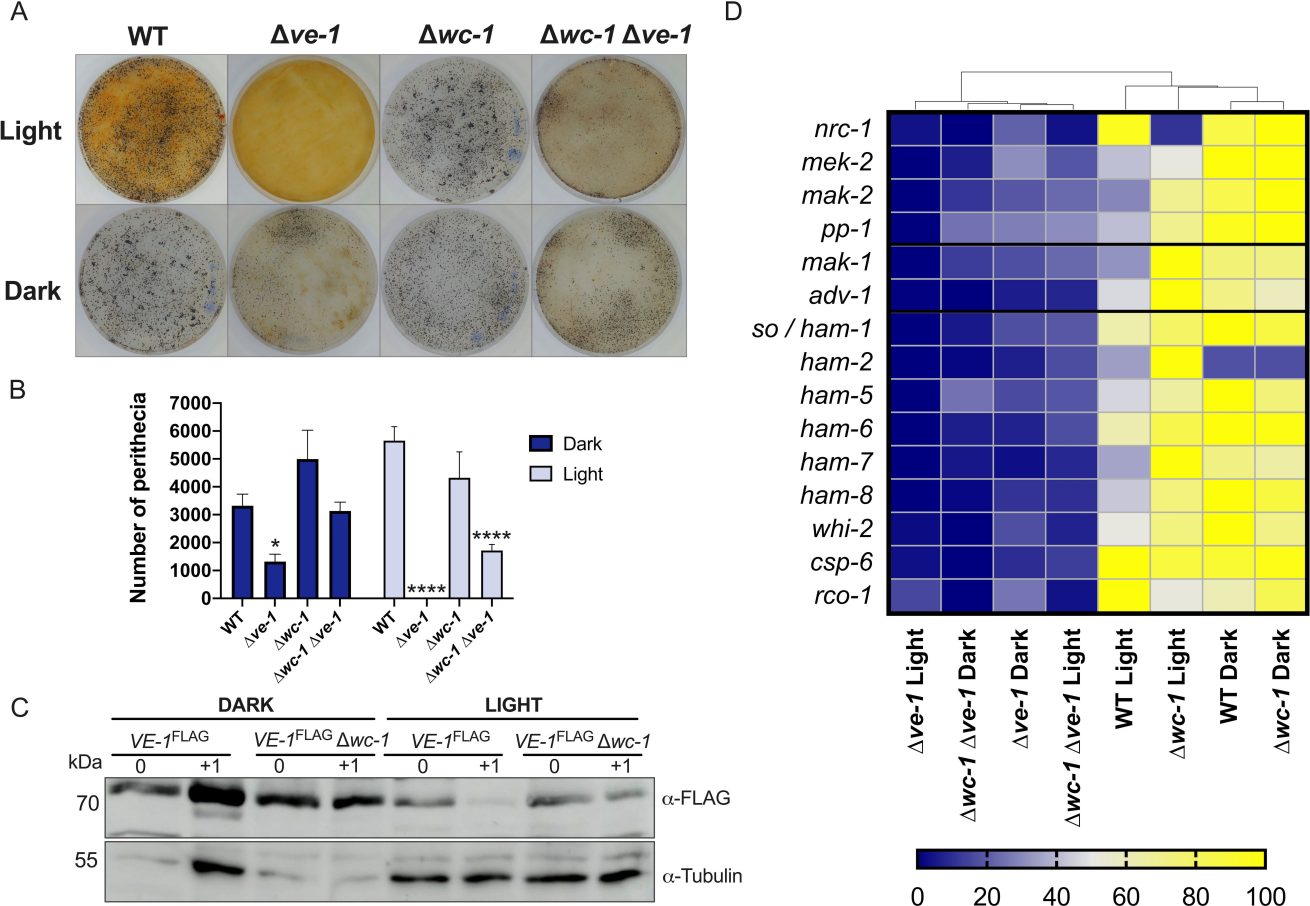
VE-1 requires the WC-1 photoreceptor for light-dependent inhibition of perithecial development. (**A**) Formation of perithecia of wild-type, ∆*ve-1,* ∆*wc-1,* and ∆*wc-1* ∆*ve-1* strains in SCM plate 7 days after fertilization in light (upper panel) and dark (lower panel). (**B**) Quantification of the total number of perithecia in plates 7 days after crossing. The plot shows the mean and standard error of 5–8 independent experiments (*P < 0.05, **P < 0.01, ***P < 0.001, ****P < 0.0001). (**C**) Accumulation of VE-1 during sexual development in the wild type and the ∆*wc-1* mutant. Protein samples from female sexual structures of strains carrying the *ve-1^FLAG^* allele before or 1 day after fertilization were separated by PAGE and hybridized with antibodies specific for the FLAG epitope or for tubulin. Forty micrograms of proteins was loaded per lane. We used tubulin as loading controls. (**D**) Heatmap showing relative transcription levels of the genes of the PR and CWI pathways misregulated by absence of VE-1 during sexual development in the dark or in the light in the wild-type, ∆*ve-1,* ∆*wc-1,* and ∆*wc-1* ∆*ve-1* strains. The hierarchical clustering of the data in the heatmap was performed with clustvis.

This observation together with the large reduction in the number of perithecia in the ∆*ve-1* strain in light suggested that WC-1, the photoreceptor component of the WCC transcription factor complex (84, 85), may participate in this regulation. Therefore, we analyzed the sexual phenotype of a double mutant with deletions in *ve-1* and *wc-1*. We observed that the light-dependent repression of sexual development in the single ∆*ve-1* mutant was lost in the double ∆*ve-1* ∆*wc-1* strain since it produced perithecia in light, unlike the single ∆*ve-1* mutant, and with a number similar to that obtained in dark (Fig. 4A and B). Accordingly, the levels of VE-1 in light increased in the ∆*wc-1* mutant (Fig. 4C).

To confirm the derepression of sexual development by the absence of WC-1 in light, we isolated RNAs from the wild-type strain, the ∆*ve-1* and ∆*wc-1* single mutants, and the ∆*ve-1* ∆*wc-1* double mutant 1 day prior to fertilization. We considered DEGs when the log_2_ of the ratio of mRNA accumulation (fold change) was greater than 1 (upregulated) or less than −1 (downregulated). The number of light-regulated genes (DEGs between light and dark) was 1,632 in the wild-type strain, and only 276 in the ∆*wc-1* mutant, as expected. However, the number of DEGs was also very reduced in the ∆*ve-1* mutant (235), and we did not detect any DEG in the double ∆*ve-1* ∆*wc-1* mutant (Data set S5). These results confirm that the absence of VE-1 results in a major alteration in the transcriptional response to light during sexual development that is additive to the absence of the photoreceptor WC-1.

The RNA-seq results showed that the absence of WC-1 in the ∆*ve-1* mutant background led to a partial recovery of the accumulation of mRNAs for the MAPK genes and other genes that participate in the MAPK signaling pathway to levels similar to the ∆*ve-1* mutant in the dark (Fig. 4D). We reasoned that in addition to the MAPK signaling pathways, other DEGs could explain the more drastic reduction in number of perithecia in the ∆*ve-1* in light. To identify those DEGs, we compared the lists from the time course experiment of ∆*ve-1* in light with the gene list showing differential expression in light between single ∆*ve-1* and double ∆*ve-1* ∆*wc-1* mutants. This approach identified 191 DEGs, whose median gene expression correlated significantly with the number of perithecia across various strains and conditions (Fig. S12). However, most of these genes are hypothetical proteins with undefined functions, indicating a likely absence of major regulatory roles. Notably, hierarchical clustering of the expression patterns of the selected genes, including the MAP kinase signaling pathways (Fig. 4D), revealed that ∆*ve-1* in light was distinct from ∆*ve-1* in the dark and the double mutant (under both light and dark conditions). This distinction is evident from its clustering outside the main cluster, consistent with the observed phenotypes (Fig. 4; Data set S5).

### Coordinated regulation of genes involved in fruiting body formation by VE-1 protein and the MAP kinases MAK-1 and MAK-2

Our results suggest that VE-1 plays a key role in regulating the mRNA levels of genes involved in both the CWI pathway and the PR pathway. These pathways, known for their interconnected nature, exhibit crosstalk between the key kinases, MAK-1 and MAK-2 (62, 86–88). Interestingly, the expression of *ve-1* and *ve-2* during protoperithecium formation remains unaffected in mutants lacking these MAP kinase genes (*mak-1* and *mak-2*) (87); this led us to hypothesize that VE-1 could act on the PR and CWI cascades, regulating directly or indirectly the expression of genes in these pathways. To explore this hypothesis further, we compared the list of genes co-regulated by MAK-1 and MAK-2 during early protoperithecia formation (87) with our data set of genes regulated by VE-1. This analysis unveiled a substantial regulatory overlap between the VE-1 and MAK-1/MAK-2 kinases. Of the 438 genes previously identified as co-regulated by MAK-1/MAK-2 (87), 265 genes displayed VE-1-dependent expression during sexual development in light (60% of the data set), 142 in the dark, and 117 genes overlapped under both conditions (Data set S6). In the set of co-regulated genes, deletion mutants in 24 genes displayed a phenotype in sexual development with diverse defects in protoperithecium or perithecium formation (50, 87). Notably, 10 single-gene deletion mutants (*pfd-1*, *oli*, NCU05948, *tcf-27*, *cea-6*, *mol-1*, *fpo-1*, *acw-5*, *gld-1*, and *eas*) were characterized as female sterile, with no perithecium formation after mating. Three of these mutants (*pfd-1*, *oli,* and *pfd-2*) shared a phenotype similar to ∆*mak-1* and ∆*mak-2* strains*,* failing to form the protoperithecium (87). In contrast, mutants with deletion of *tcf-27*, *cea-6,* and *mol-1* showed a reduction in protoperithecia formation. The remaining four genes were not necessary for protoperithecia formation, but their corresponding mutants exhibited defects in protoperithecia maturation, mating, or perithecia development (87). Our results strongly suggest a coordinated regulatory role among VE-1, MAK-1, and MAK-2 in the transcriptional control of perithecium development.

### The VE-1/VE-2 protein complex binds to the promoters of MAP kinase genes of the PR, CWI, and OS signaling pathways

**Fig 5:**
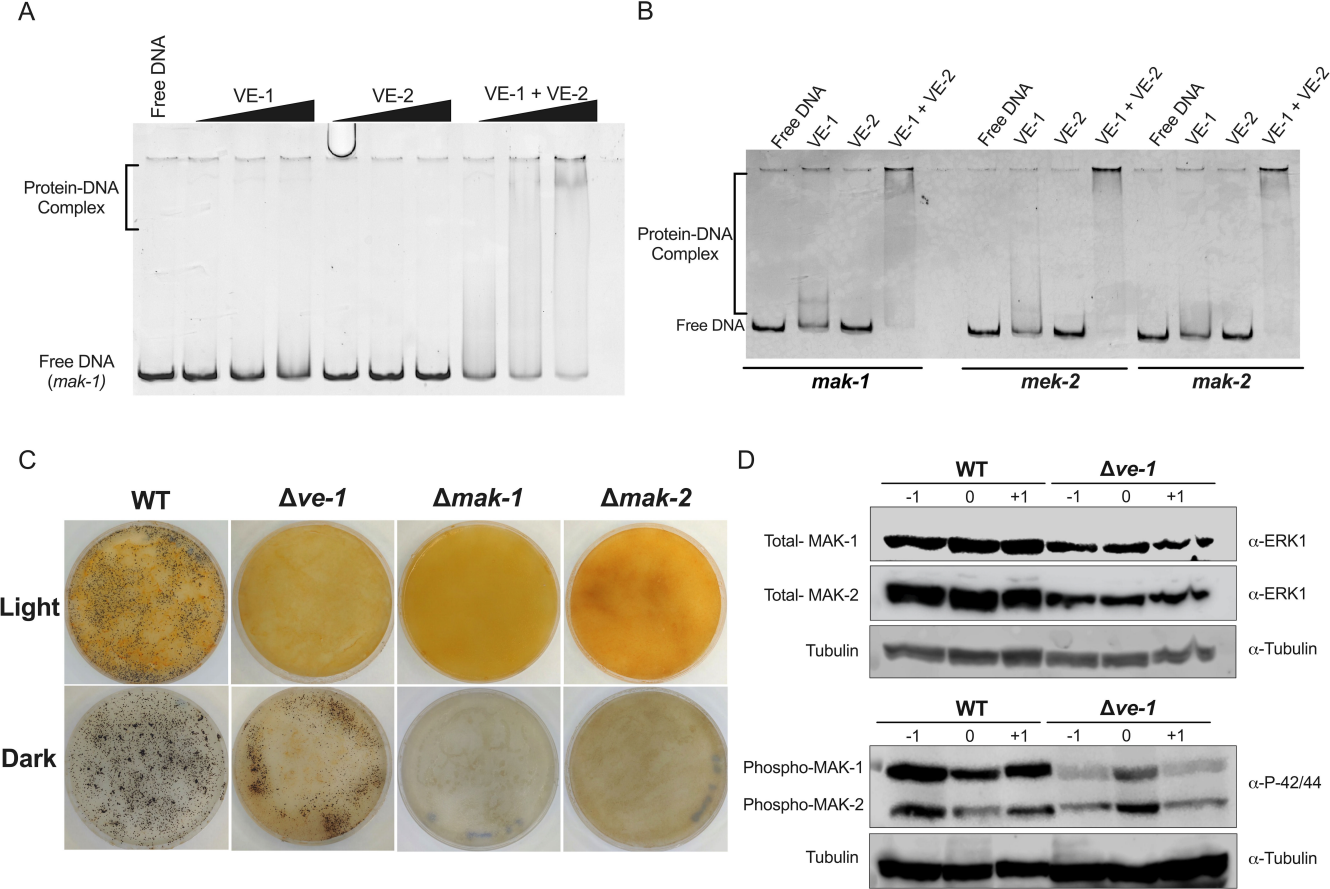
VE-1 regulation of MAPK signaling components. (**A**). EMSA of the *mak-1* promoter employing 0.3, 0.6, or 1 µM of each protein (VE-1, VE-2, or VE-1 + VE-2) and 150 ng of the DNA probe. (**B**) EMSA of promoters from the MAP kinase genes misregulated in the ∆*ve-1* strains. The EMSA experiment was performed with 150 ng of DNA probe (1 kb of the promoter region of *mak-1, mek-2,* and *mak-2*) and 1 µM of each protein in each reaction. (**C**) Formation of perithecia of wild-type, ∆*ve-1,* ∆*mak-1,* and ∆*mak-2* strains in SCM plate 7 days after fertilization in light (upper panel) and dark (lower panel). (**D**) Accumulation of proteins for the components of the MAPK cascades MAK-1 and MAK-2 during sexual development. Protein samples from sexual cultures of wild type and ∆*ve-1* strains before or after fertilization at day 0 were separated by PAGE. Membranes were hybridized antibodies against MAP kinase (ERK-1, ERK-2), phospho P44/42 MAPK and tubulin. Forty micrograms of proteins was loaded per lane.

The velvet domain of the VosA protein of *A. nidulans* is an RHD-like domain with homology to NF-κB transcription factors that binds to an 11-nucleotide sequence (27). Since VE-1 contains a velvet domain, it is tempting to speculate that VE-1 might regulate the formation and maturation of perithecia by directly binding to the promoters of regulatory genes during sexual development. We performed electrophoretic mobility shift assays (EMSA) to assess whether the proteins VE-1, VE-2, and the heterodimer VE-1/VE-2 produced in *E. coli* cells could bind to the promoters of a set of genes regulated by VE-1. VE-1 or VE-2 where expressed in *E. coli* cells, the proteins were purified, and then used either separately or mixed in each EMSA. We selected a set of MAP kinase genes that regulate sexual development: *mek-2* and *mak-2* (PR pathway) and *mak-1* (CWI pathway). We observed binding of VE-1, but not VE-2, to the promoters of these three MAPK genes. DNA binding was more pronounced when both VE-1 and VE-2 were included in the reaction, indicating a higher DNA-binding affinity for the VE-1/VE-2 heterodimer (Fig. 5A and B). Our results confirm that VE-1/VE-2 binds to the promoters of a set of MAPK genes and support our proposal that the velvet protein complex regulates transcription of these genes by direct binding to promoters, at least for the set of genes analyzed.

### VE-1 regulates the accumulation and phosphorylation of MAK-1 and MAK-2

The changes in the transcriptome observed in the ∆*ve-1* mutant suggested that the absence of VE-1 would lead to changes in the accumulation of key regulators of sexual development, including several MAPK. We assayed the accumulation of MAK-1 and MAK-2 by western blot in both the wild type and the ∆*ve-1* mutant during the early stages of sexual development (Fig. 5D; Fig. S13). Absence of VE-1 led to a decrease in the accumulation of MAK-1 and MAK-2 when compared with the wild type. In addition, we observed a reduction in the accumulation of phosphorylated MAK-1 in the mutant across all developmental stages, but the reduction in phosphorylated MAK-2 was mainly observed 1 day before fertilization. Sexual development in the ∆*ve-1* mutant is mostly absent in strains incubated in the light, but the absence of sexual development was observed in strains with deletions of *mak-1* or *mak-2* cultured in dark or light (Fig. 5C). Our results suggest that VE-1 is required for the accumulation and regulation of MAK-1 and MAK-2 and that the alterations of sexual development observed in the ∆*ve-1* mutant were, in part, a consequence of the misregulation of the MAPK pathways.

## DISCUSSION

The development of sexual structures in fungi requires cellular differentiation and morphogenesis. This process is regulated by a complex cascade of receptors and signal transduction components that lead to changes in transcriptional regulation (5, 30). The velvet protein complex plays a crucial role in integrating extracellular signals with transcriptional regulation during fungal development. In *A. nidulans*, the founding member of the family, the VeA protein interacts with another velvet protein, VelB, positively regulating the formation of sexual structures (33, 89). The role of the VeA/VelB complex in the regulation of sexual development appears to be conserved. Apart from studies in Eurotiomycetes, homologs of VeA were found to positively regulate female fertility in species of Leotiomycetes (65, 90) and Sordariomycetes (36, 38, 91), including *N. crassa*, as reported here. However, the role of LaeA/LAE-1 in sexual development does not seem to be as well conserved. The phenotype of the deletion of *laeA* homologs varies from mild or no reduction in the number of sexual structures to complete absence, depending on the species (Fig. 1) (32, 41, 90). Despite the conservation of the role of velvet proteins, the regulation of the velvet dimeric complex differs between species. In *A. nidulans*, the coordination of sexual and asexual development depends on the subcellular localization of VeA, which is regulated by light (33, 89). In *N. crassa*, light regulates the accumulation of VE-1 during asexual development, and accumulation of VE-1 and VE-2 during sexual development, but not the abundance of LAE-1 (Fig. 1) (40, 41). The absence of VE-1 or VE-2 in light (Fig. 1C) was puzzling considering the light-dependent phenotype of the corresponding mutants. However, we observed VE-1 in cultures exposed to light after an optimized hybridization procedure (Fig. 4C), an indication that the reduced amount of VE-1, and possibly VE-2, in cultures exposed to light was sufficient for its regulatory role. Indeed, deletion of *wc-1* increased the amount of VE-1 in light and partially reverted the ∆*ve-1* phenotype, supporting the hypothesis that control of the VE-1 levels is necessary for the regulation of sexual development.

As expected for a master regulator, the deletion of the *ve-1* gene affects the expression of thousands of genes, many of which are also involved in other morphogenetic programs, similar to what was found in aspergilli (92, 93). The differences in gene expression between the wild type and the ∆*ve-1* mutant strains were found to be larger in light than in darkness, consistent with the more drastic reduction in perihecia formation found in light. The set of genes whose expression was dependent on VE-1 was enriched in genes involved in signal transduction and cell regulation. In particular, we observed the downregulation of the complete PR/MAK-2 MAP kinase signaling pathway in the ∆*ve-1* mutant, especially in light conditions, affecting transcript, and total and phosphorylated protein levels (Fig. 3 to 5). The PR MAP kinase is highly conserved across Eukarya, and in fungi, it is involved in the regulation of cell fusion, pheromone-induced mating, and the formation of fruiting bodies (54, 55, 88, 94). Deletion of any of the core genes of the PR/MAK-2 MAP kinase pathway blocks mating fungi, including many human and plant pathogens, such as the Ascomycetes *S. cerevisiae* (95, 96), *A. nidulans* (97) and *A. flavus* (98, 99), *Candida albicans* (100, 101), and *F. graminearum* (102, 103), and Basidiomycetes, such as *Cryptococcus* (104, 105) and *Ustilago maydis* (106, 107). The PR MAP kinase is widely distributed in the fungal kingdom, extending also to early diverging fungi where expansion of signal transduction gene families has been observed (Fig. S14), as previously reported (108).

A connection between vegetative hyphal fusion genes (VHF) and sexual development has been proposed, as several mutants defective in cell fusion were unable to produce fertilized perithecia (56, 57, 61, 78, 109). The expression of several cell fusion genes was downregulated in the ∆*ve-1* mutant during sexual development, regardless the light condition (*ham-6, ham-7, ham-8,* and *whi-2),* whereas some other were significantly downregulated only when cultures were exposed to light (e.g. *so/ham-1, ham-2, ham-5,* and *mob-3*). Interestingly, all these genes are known to participate in signal transduction as components of both CWI/MAK-1 and PR/MAK-2 pathways. Considering that VE-1 contains a structurally similar DNA binding domain to the mammalian TF NF-κB, it is reasonable to think that VE-1 could regulate transcription by binding directly to the promoters of the target effector genes, as previously demonstrated for the velvet proteins VosA (27) and VeA (93, 110). We have detected *in vitro* binding of VE-1 and the heterodimer VE-1/VE-2 to the promoter sequences of *mak-1*, *mak-2,* and *mek-2*. VE-2 alone does not bind to the DNA regions tested, but the binding of VE-1 is improved by the presence of VE-2. These results suggest a main role for VE-1 in the molecular mechanism of DNA binding by the velvet complex VE-1/VE-2 and that VE-2 plays an accessory role in improving the affinity of the complex for DNA binding, serving as direct regulators of the expression of MAP kinase signaling genes. The alterations in the amount and activity of the kinases MAK-1 and MAK-2 observed in the ∆*ve-1* strain support the proposal that VE-1 regulates the development of sexual structures through a direct regulation of transcription and the activity of the MAPK pathways. Indeed, deletion of the MAP kinase genes *mak-1* or *mak-2* blocked the formation of perithecia, regardless of the light condition, indicating that the light-dependent sexual phenotype is determined by VE-1. Given the wide distribution of velvet proteins in the fungal kingdom (28), and the conservation of the MAPK signaling pathways during sexual development, it is tempting to speculate that velvet proteins regulate sexual development in other fungi by a mechanism similar to the one that we have described for *N. crassa*. Indeed, VeA was found to bind to the promoter of the *mak-2* homolog (*mpkB*) in *A. nidulans* (93).

MAK-2 homologs participate in the regulation of cellular morphogenesis and differentiation across a broad variety of organisms, ranging from yeasts to humans. This suggests that they were likely present in the last common ancestor of fungi and were potentially involved in regulating fungal mating. Velvet proteins have been reported to be developmentally regulated in Pezizomycotina and Agaricomycotina (111) and may have allowed fungi to integrate intricate environmental signals for the precise regulation of fruiting body formation by modulating the levels and activity of MAP kinase signaling pathways.

In summary, our observations support the proposal that VE-1 and VE-2 form a heterodimer protein complex that regulates sexual development by controlling the expression of the PR-MAP kinase signaling pathway and other key genes involved in cell communication and hyphal fusion, including transcription factor genes.

## MATERIALS AND METHODS

### Strains and culture conditions

Fungal strains used in this work are listed in Table S1. *E. coli* DH5α was used for plasmid manipulations. Strain manipulation and growth media preparation followed standard procedures and protocols. General procedures and media for *N. crassa* are available at the Fungal Genetic Stock Center website (https://www.fgsc.net/Neurospora/NeurosporaProtocolGuide.htm).

Induction of protoperithecia development was carried out by spreading a total of 2 × 10^4^ conidia into nitrogen-poor SCM agar plates (81) and then incubated at 22°C in the light or in the dark, depending on the experiment, for 7 days to allow the formation of protoperithecia. Conidia from a strain of opposing mating type were collected and suspended in 2.5% Tween 20. Formation of protoperithecia in each strain was examined using a stereomicroscope between days 5 and 7. Fertilization was performed by applying 2 mL of a conidia suspension (5 × 10^5^ conidia/mL in 2.5% Tween 20) to the surface of the plates containing protoperithecia. After fertilization, sexual development occurred under the same culture conditions as protoperithecium development, and all the process was monitored using a stereomicroscope. Fungal material was harvested by scrapping the surface with a razor blade. Samples were collected 1 and 2 days prior to fertilization, immediately before fertilization (day 0) and 1, 2, 4, and 6 days after fertilization. All tissues and perithecia were immediately frozen in liquid nitrogen and stored at −80°C.

Constant illumination was provided by a set of fluorescent lamps (containing 1 W/m^2^ of blue light). All the manipulations in the dark were performed under red light. At least three independent biological replicates were employed in RNA or protein quantification experiments.

### Optical and scanning electron microscopy

Samples for optical microscopy were taken from liquid or solid cultures depending on the experiment, placed on a slide, and visualized with a Leica EC3 instrument. Samples for stereomicroscopy were visualized with Leica M205FCA. For image processing, we used LASX 3.7.4.23463 software.

For SEM strains were inoculated on Vogel solid medium and cultured for 24 h at 34°C. Samples were prepared as previously described (112). Briefly, small squares of the solid cultures were cut and placed on 10 mL polypropylene tubes. Samples were fixed in 2.5% glutaraldehyde and post-fixed in 1% osmium tetroxide. Samples were freeze-dried for 12–18 h and finally coated with gold. Samples were visualized with a JEOL 6460LV instrument.

### RNA isolation for transcriptomic sequencing

For the analysis of perithecia formation throughout sexual development, strains of complementary mating type, wild-type *mat a*, wild-type *mat A*, and ∆*ve-1 mat A* (Table S1) were used. Induction of the formation of protoperithecia and then maturation of perithecia after fertilization were performed as described in the culture conditions section. Samples were collected 1 and 2 days prior to fertilization, immediately before fertilization (day 0), and 1, 2, 4, and 6 days after fertilization. In addition, transcriptome comparisons between wild type, ∆*wc-1*, ∆*ve-1*, and the double mutant ∆*wc-1* ∆*ve-1* 1 day prior fertilization were performed. For transcriptomic analysis, all tissues were immediately frozen in liquid nitrogen and stored at −80°C. Tissue samples from biological replicates were pooled for RNA extraction at each sample point. Mycelium was ground using a precooled mortar and pestle, homogenized with Tri Reagent (AM9738, Invitrogen), and then phase-separated by adding chloroform. The nucleic acid phase was precipitated with isopropanol, washed with 75% ethanol, and solubilized in RNase-free water. RNA clean-up and DNase treatment were performed using Nucleo-Spin RNA (Macherey-Nagel) and RNase-free DNase Set (Qiagen), following the manufacturer’s procedures. RNA quantity and quality were analyzed using Nano-drop ND-1000 spectrophotometer and agarose gel electrophoresis. The RNA samples were stored at −80°C before use.

### cDNA library preparation and statistical analysis

The mRNA quality control and the preparation of the strand-specific cDNA library was performed according to the Illumina mRNA sample preparation guide. Sequencing was performed on Illumina Genome Analyzer (Illumina NovaSeq 6000) at the Next Generation Sequencing Facility Novogene.

The quality of the reads was checked using FastQC (v0.11.9) (http://www.bioinformatics.babraham.ac.uk/projects/fastqc/). The reads were cleaned with fastp (v0.23.4) using default parameters. Pair-end reads of 150 nucleotides each (first-strand PE150) were mapped against the *N. crassa* OR74A genome (NC12.37) obtained from JGI mycocosm web portal https://mycocosm.jgi.doe.gov/Neucr2/Neucr2.home.html using STAR (v2.7.10b). Subsequently, the BAM files generated during the mapping phase were quantified with the featureCounts command of the Subread package taking into account the type of library (first-strand). In this last step, the raw reads file was generated. From this point on, two different protocols were followed, one for time series analysis and the other for pairwise comparisons between experimental conditions.

The time series analysis was performed with maSigPro (v1.74.0) both in its “single” mode (with one experimental condition) and in its “multiple” mode (comparison of two experimental conditions) (49). maSigPro requires a prior normalization of the raw reads for which we used edgeR (v4.0.3) using TMM (trimmed mean of M values) normalization. To fit the time series with maSigPro, we used a quadratic regression model with the settings Q = 0.001, α = 0.001, and *R*-squared = 0.7. Significant genes were distributed into nine clusters using the “hclust” method.

Pairwise comparisons were performed with DESeq2 (v1.42.0) using default parameters (113). In general, a gene was considered to be differentially expressed when the adjusted *P* of the Wald test for multiple testing was less than 0.05 and whose log2FC was greater than 1 (upregulated) or less than −1 (downregulated). However, more restrictive criteria were used in some comparisons and are indicated in each results section.

Genes showing significant differences in their expression profile (DEGs) were classified according to their known or putative gene information in the integrated functional genomic database Fungi DB (114). All the genes selected were used to create Venn diagrams using https://bioinformatics.psb.ugent.be/webtools/Venn/, followed by GO analysis using Fungi DB database for the enrichment of GO terms into biological processes. The significantly associated gene ontology terms (adjusted *P*-value < 0.05) were imported to Revigo, where they were clustered based on their relatedness, and any redundancy was removed (87). Heat maps were generated with counts per million (CPM) values using GraphPad Prism 8.2.1 (Macintosh version) (GraphPad Software, San Diego, CA, USA, https://www.graphpad.com/).

### Protein isolation and detection

Proteins were extracted from mycelia by previously described methods (40) using a modified lysis buffer (50 mM HEPES [pH 7.4], 137 mM NaCl, 10% glycerol, 5 mM EDTA, 29.3 µM phenylmethylsulfonyl fluoride [PMSF], 6.3 µM leupeptin, 4.4 µM pepstatin A). Total proteins were subjected to SDS-PAGE on 10%–12% (29:1) acrylamide-bisacrylamide gels and transferred to nitrocellulose membranes. Equal loading was confirmed by staining the hybridization membrane with Ponceau S solution. Membranes were hybridized with monoclonal antibodies against FLAG (F3165; 1:10,000, Sigma), HA (901513; 1:5,000, Biolegend), and tubulin (sc-32293, 1:500 Santa Cruz) or polyclonal antibodies against MAP kinase (ERK-1, ERK-2) (M5670 1:10,000; Sigma), and against phospho P44/42 MAPK (#9101; 1:1,000 Cell Signaling). Horseradish peroxidase (HRP)-conjugated goat α-mouse IgG (1721011, 1:10,000; Bio-Rad) and goat α-rabbit IgG (1706515, 1:10,000; BioRad) were used as secondary antibodies. Detection was performed with a chemiluminiscent-HRP substrate (BioRad 170-5060) for all western blots, except for Fig. 4C, where we used Merck Millipore WBKLS0100.

### Cloning, expression and purification of VE-1 and VE-2 proteins

For overproduction and purification of proteins VE-1 and VE-2, we used the Intein-Mediated Purification with an Affinity Chitin-binding Tag (IMPACT) system (New England Biolabs). The mRNAs of *ve-1* and *ve-2* were cloned into the protein-generating vector pTXB1 after RT-PCR amplification (Table S2), which allowed the fusion of the cleavage intein tag to the C-terminus of the target protein. The resulting plasmids bearing the target genes were transformed into competent cells prepared from *E. coli* ER2566. Cell culture, protein induction, and purification (including self-cleavage to eliminate the tag) were performed following manufacturer’s instructions. Protein induction was optimized by testing different induction times and different culture temperatures. To concentrate the samples, 30 kDa centricons (Milipore) were used, centrifuging 4,000 × *g* at 4°C until reaching the desired volume. Protein dialysis was performed using the Slide-A-Lyzer dialysis system (Thermo Scientific) following the manufacturer’s instructions. Dialysis was performed at 4°C for 16 h in dialysis buffer (25 mM HEPES [pH 7.4], 250 mM NaCl, 5% glycerol, and 2 mM DTT) with shaking.

### Electrophoretic mobility shift assay

DNA probes for the protein-nucleic acid interaction analysis were generated by PCR and primers listed in Table S2, using the Q5 High-Fidelity polymerase system (New England BioLabs). Binding reactions were performed in a 15 µL reaction volume containing 3 µL of 5× binding buffer (125 mM HEPES, 250 mM NaCl, 10 mM DTT, and 15% [vol/vol] glycerol), ≅100 ng of DNA probe, and the appropriate amount of each purified protein. The mixture was incubated for 20 min at room temperature, and then, the reaction was stopped by adding 3 µL of 6× loading buffer (1 mM EDTA, 10 mM Tris HCl [pH 8], 30% [vol/vol] glycerol, 0.125% bromophenol blue [wt/vol], and 0.125% [wt/vol] xylene cyanol) to reach a final volume of 18 µL. To analyze the DNA-protein reaction, the mixtures were subjected to electrophoresis in 5% (wt/vol) acrylamide native gel in TBE buffer (90 mM Tris-boric acid, 2 mM EDTA [pH 8.3]) at 4°C. Gels were stained with ethidium bromide for 10 min and subsequently visualized in an ultraviolet transilluminator.

## Data Availability

RNAseq results have been deposited in the Gene Expression Omnibus (GEO) database under accession number GSE224796 (RNAseq of a time-course of sexual development of the wild-type and ∆*ve-1* strains), and GSE252195 (RNAseq of sexual development (time −1) of the wild-type, ∆*ve-1,* ∆*wc-1,* and ∆*wc-1* ∆*ve-1* strains).

## SUPPLEMENTAL MATERIAL

Data Set S1A mbio.02264-24-s0001.xlsx RNAseq of the wild type during sexual development.

Data Set S1B mbio.02264-24-s0002.xlsx RNAseq of the ∆*ve-1* mutant during sexual development.

Data Set S2 mbio.02264-24-s0003.xlsx Characterization of the regulation of transcription during sexual development of *N. crassa* wild-type and Δ*ve-1* strains.

Data Set S3 mbio.02264-24-s0004.xlsx Genes regulated by VE-1 during sexual development.

Data Set S4 mbio.02264-24-s0005.xlsx Genes regulated by VE-1 required for sexual development.

Data Set S5 mbio.02264-24-s0006.xlsx Genes regulated in the Δ*ve-1*, Δ*wc-1*, and Δ*ve-1* Δ*wc-1* mutant strains in sexual development.

Data Set S6 mbio.02264-24-s0007.xlsx Coordinated regulation of genes involved in fruiting body formation by VE-1 protein, MAK-1, and MAK-2.

Supplemental material mbio.02264-24-s0008.pdf Figures S1-S14, Tables S1 and S2, and data set legends.

ASM does not own the copyrights to Supplemental Material that may be linked to, or accessed through, an article. The authors have granted ASM a non-exclusive, world-wide license to publish the Supplemental Material files. Please contact the corresponding author directly for reuse.
