# Supplementary material for: VE-1 regulation of MAPK signaling controls sexual development in Neurospora crassa
Source: mBio. 2024 Sep 16;15(10):e02264-24. doi: 10.1128/mbio.02264-24 (PMC11481897; doi:10.1128/mbio.02264-24)
Supplement: Supplemental material — Figures S1-S14, Tables S1 and S2, and data set legends. [file mbio.02264-24-s0008.pdf]

SUPPLEMENTARY MATERIAL

Supplementary figures

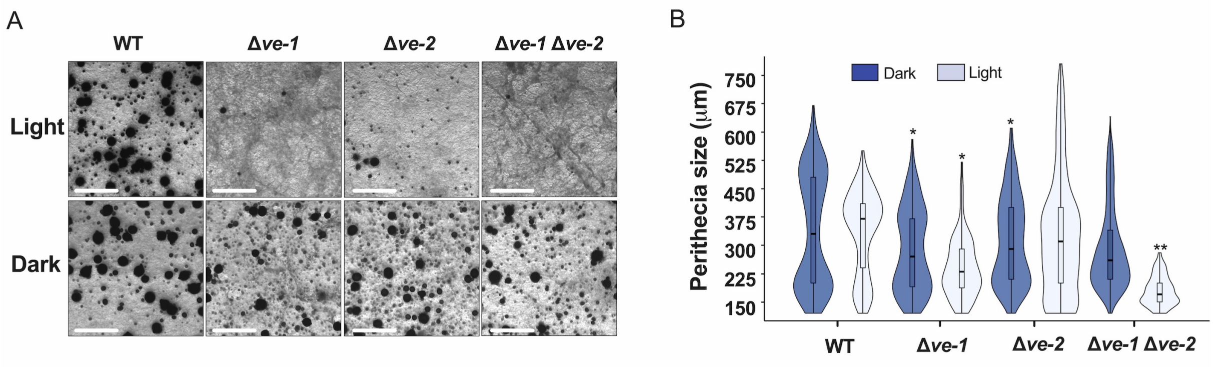

Fig. S1. Characterization of the morphology and size of the perithecia in velvet mutants. (A) Microscope images showing perithecia developed after seven days post-fertilization in the wild-type strain and single and double Δ*ve-1* and Δ*ve-2* mutants. Scale bar = 2 mm. (B) Measurement of the size of the perithecia population in the wild-type strain and single and double Δ*ve-1* and Δ*ve-2* mutants. The asterisks show significance with a two-way ANOVA test (* $p< 0.05$, ** $p<0.01$).

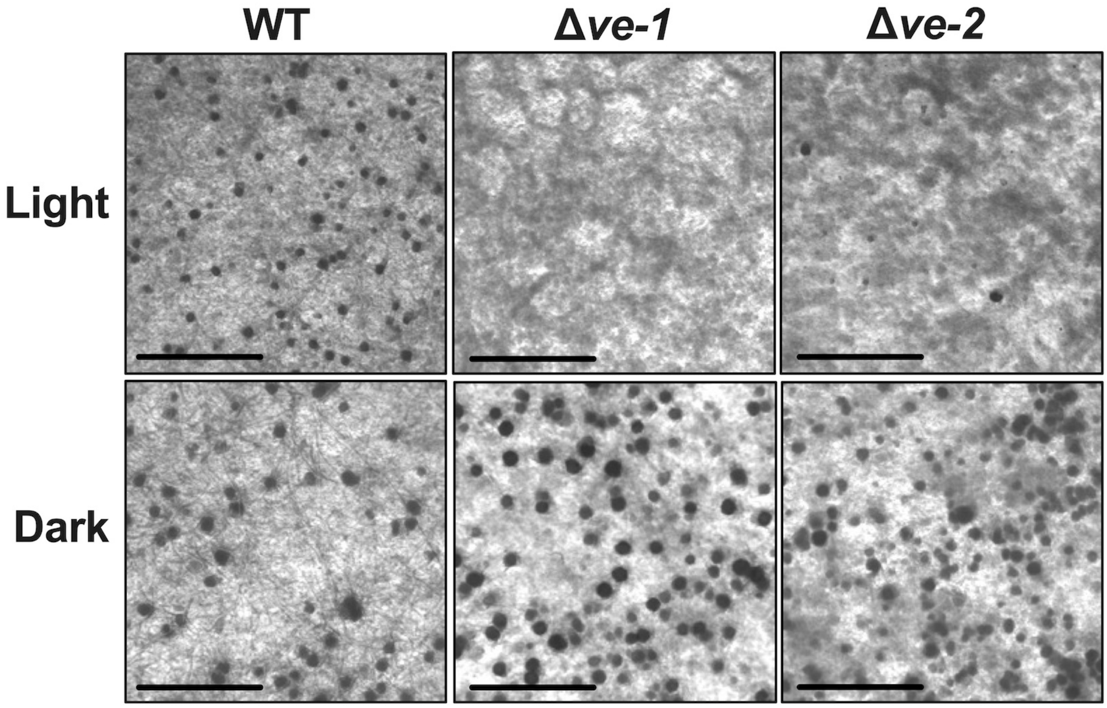

Fig S2. Light dependent inhibition of protoperithecia formation in the velvet mutants. Formation of protoperithecia in the wild-type, Δ*ve-1* and Δ*ve-2* strains. A total of 2x10^4 conidia were spread on SCM plates and incubated for seven days at 22°C under both dark and light conditions prior to observing the development of protoperithecia. Scale bar = 1mm.

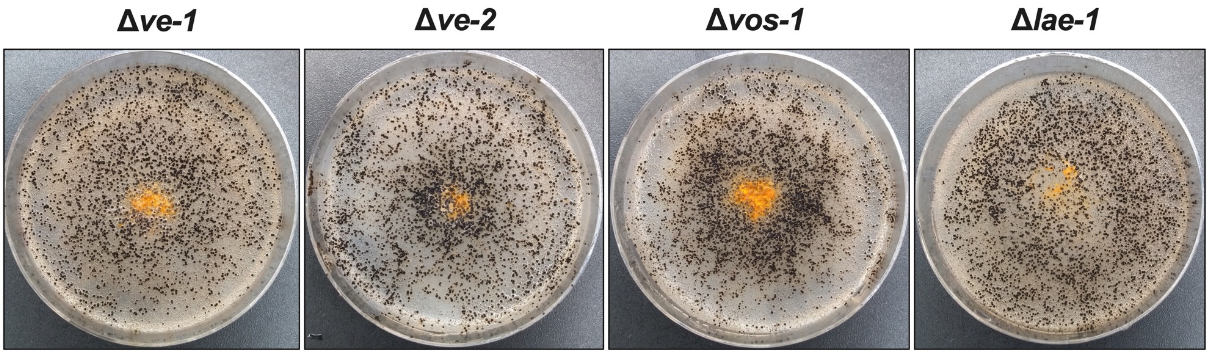

Fig S3. VE-1, VE-2, VOS-1, and LAE-1 are not required for male fertility during sexual development. Plates inoculated with 5 µl of a wild-type conidia suspension (10^4 conidia/ml) were incubated for seven days at 22°C to allow protoperithecia formation and were covered with a suspension of 10^6 conidia from mutant strains. The plates were incubated for a minimum of seven additional days under the same conditions until perithecia matured and ascospores were released. The plates were washed with ethanol to remove the aerial hyphae covering the plates prior to imaging.

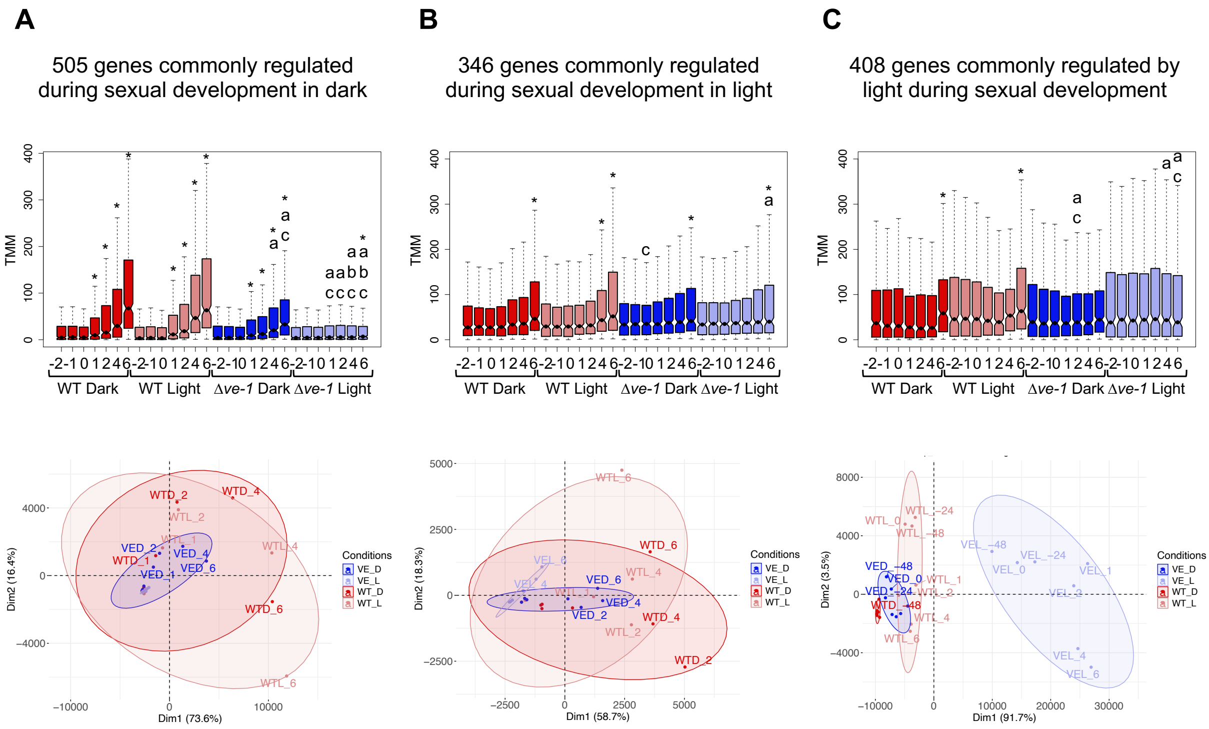

Fig S4. Expression pattern of the genes commonly regulated in the wild-type strain and the Δ*ve-1* mutant. Boxplot and PC analysis of the **(A)** 505 commonly regulated during sexual development in the dark, **(B)** 346 commonly regulated during sexual development in the light, and **(C)** 408 commonly regulated by light during sexual development between the wild type and the Δ*ve-1* mutant reported in Fig. 2C. The boxplots show the median expression of the genes (TMMs) in each condition. In both the boxplots and the PC analysis graphs, blue depicts the Δ*ve-1* in the dark, light blue the Δ*ve-1* in the light, red the wild type in the dark, and light red the wild type in the light. “a” denotes that values for the wild type are significantly different from those for the Δ*ve-1* mutant in the same conditions (light/dark and days) with a two-sample Mann-Whitney U Test ($p<0.05$) and “b” with Vargha and Delaney’s A ($A >0.71$). “c” denotes that the median of the wild type and the median of the Δ*ve-1* mutant in the same conditions are not equal according to Mood’s median test ($p<0.01$). Asterisks denote that values at each time point are significantly different from those at the first time point (-2: two day prior to fertilization) for each strain in the same conditions (light/dark and days) with a two-sample Mann-Whitney U Test ($p<0.01$).

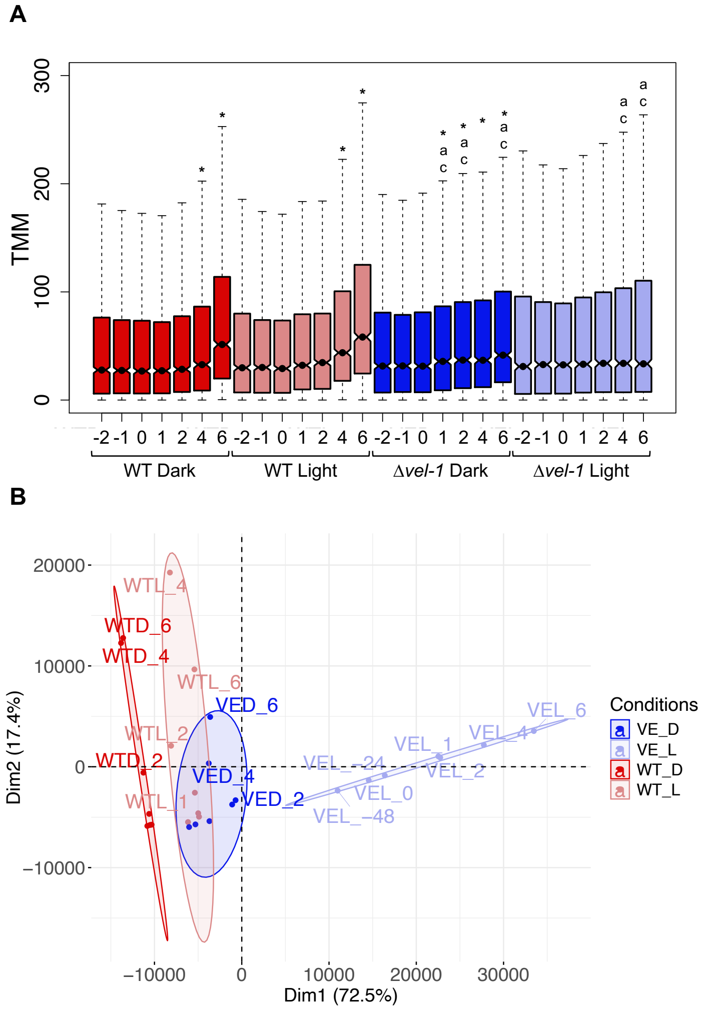

Fig S5. Expression pattern of the genes commonly regulated in the wild-type strain and the Δ*ve-1* mutant. (A) Boxplot and **(B)** PC analysis of the 1646 DEGs commonly regulated during sexual development in the dark and in the light reported in Fig. 2D. The boxplots show the median expression of the genes (TMMs) in each condition. In both the boxplots and the PC analysis graphs, blue depicts the Δ*ve-1* in the dark, light blue the Δ*ve-1* in the light, red the wild type in the dark, and light red the wild type in the light. "a" denotes that values for the wild type are significantly different from those for the Δ*ve-1* mutant in the same conditions (light/dark and days) with a two-sample Mann-Whitney U Test ($p<0.05$) and "b" with Vargha and Delaney's A (A >0.71). "c" denotes that the median of the wild type and the median of the Δ*ve-1* mutant in the same conditions are not equal according to Mood's median test ($p<0.01$). Asterisks denote that values at each time point are significantly different from those at the first time point (-2: two day prior to fertilization) for each strain in the same conditions (light/dark and days) with a two-sample Mann-Whitney U Test ($p<0.01$).

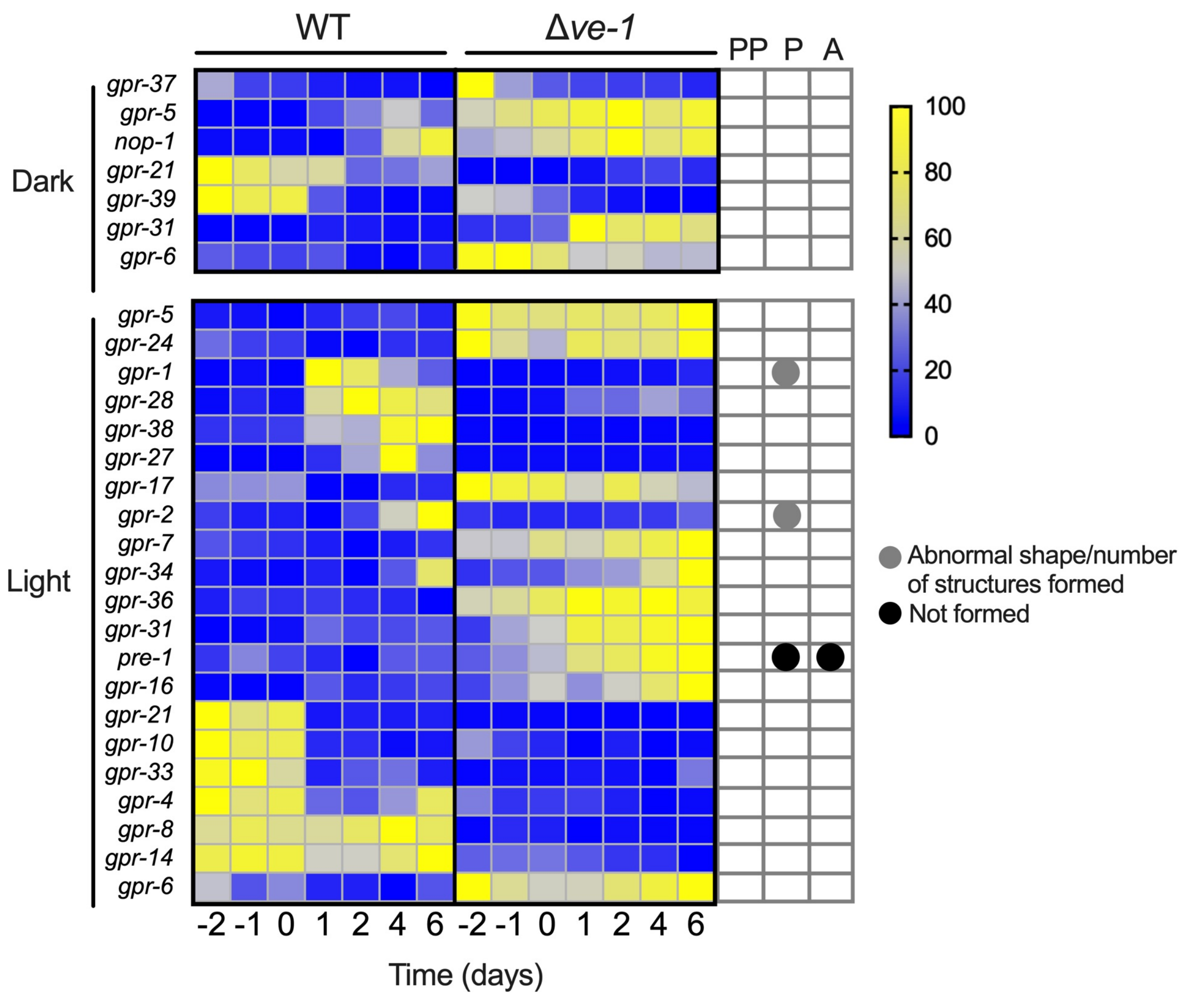

Fig. S6. GPCRs encoding genes differentially expressed in the Δ*ve-1* mutant strain. Heatmap showing relative normalized transcription levels (TMMs) of genes misregulated by absence of VE-1 during sexual development in the dark (upper panel) or in the light (lower panel). Protoperithecia were fertilized at time 0. DEGs were identified after the analysis performed by magSigPro. *gpr-5, gpr-6*, *gpr-21*, and *gpr-3*1 are misregulated in dark and light. The table on the right side of the figure is a summary of the phenotype for the mutants lacking each GPCR. The gray circles denote that the indicated structure shape or number of structures formed is abnormal compared to the WT, while black circles show that the indicated structure is not formed. The absence of a circle indicates that there was no defect observed in sexual development. PP, Protoperithecia; P, Perithecia; A, Ascospores. Phenotype data were obtained from (1).

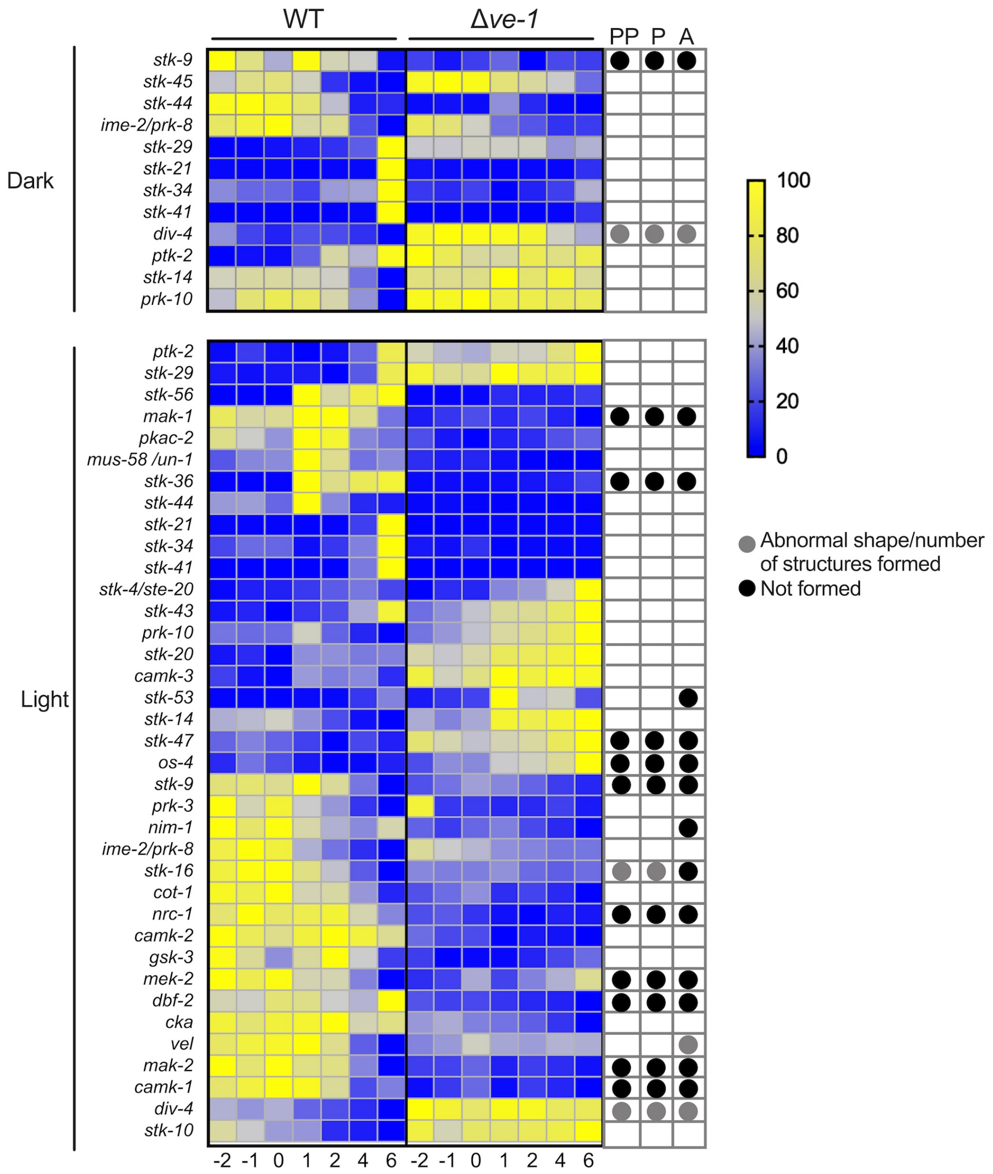

Fig. S7. Transcriptomic profiles of genes encoding S/T kinases during sexual development. Heatmap showing relative normalized transcription levels (TMMs) of genes misregulated by absence of VE-1 during sexual development in the dark (upper panel) or in the light (lower panel). Protoperithecia were fertilized at time 0. DEGs were identified after the analysis performed by magSigPro. Kinases *stk-9*, *ime-2* and *stk-14* were misregulated in dark and light. The table on the right side of the figure is a summary of the phenotype for the mutants lacking each kinase. The gray circles denote that the indicated structure shape or number of structures formed is abnormal compared to the WT, while black circles show that the indicated structure is not formed. The absence of a circle indicates there was no defect observed in sexual development. PP, Protoperithecia; P, Perithecia; A, Ascospores. Phenotype data were obtained from (1).

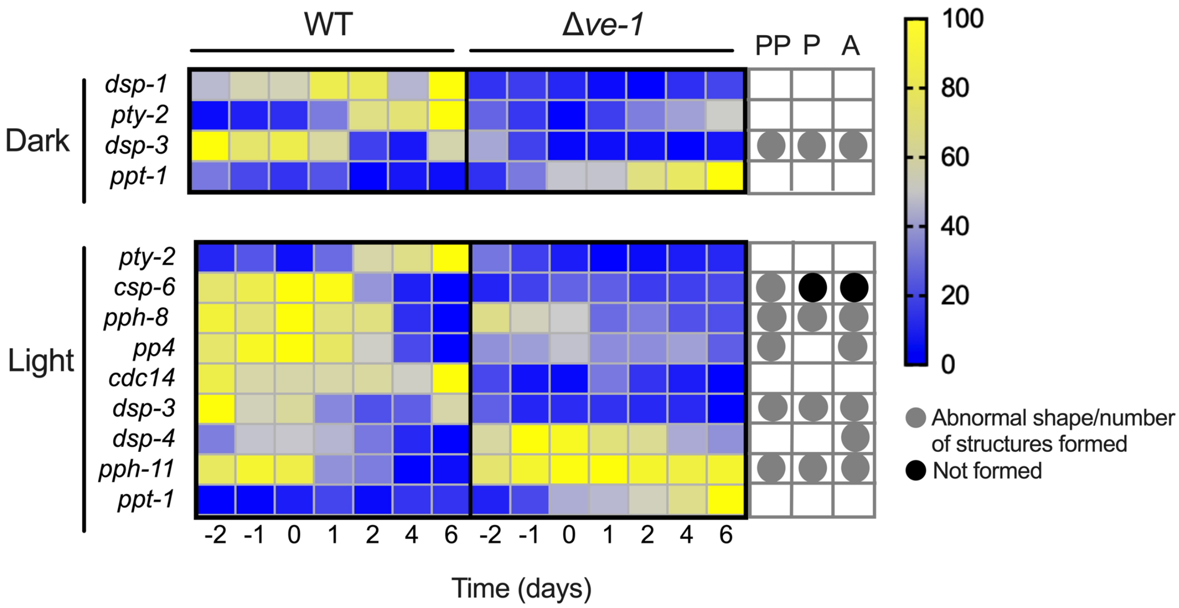

Fig. S8. Transcriptomic profiles of genes encoding S/T phosphatases during sexual development. Heatmap showing relative normalized transcription levels (TMMs) of genes misregulated by absence of VE-1 during sexual development in the dark (upper panel) or in the light (lower panel). Protoperithecia were fertilized at time 0. DEGs were identified after the analysis performed by magSigPro. Phosphatases *pty-2, dsp-3* and *ppt-1* were misregulated in dark and light. The table on the right side of the figure is a summary of the phenotype for the mutants lacking each phosphatase. The gray circles denote that the indicated structure shape or number of structures formed is abnormal compared to the WT, while black circles show that the indicated structure is not formed. The absence of a circle indicates there was no defect observed in sexual development. PP, Protoperithecia; P, Perithecia; A, Ascospores. Phenotype data were obtained from (1).

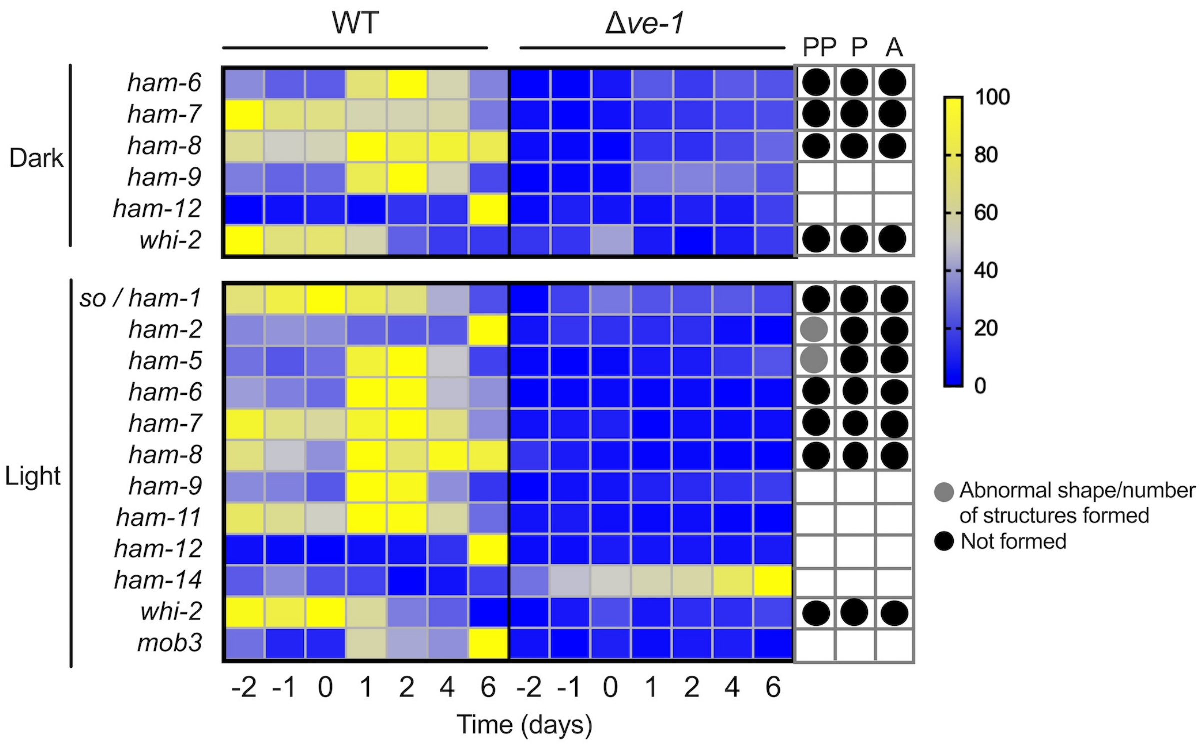

Fig. S9. Transcriptomic profiles of cell-to cell fusion genes during sexual development. Heatmap showing relative normalized transcription levels (TMMs) of genes misregulated by absence of VE-1 during sexual development in the dark (upper panel) or in the light (lower panel). Protoperithecia were fertilized at time 0. DEGs were identified after the analysis performed by magSigPro. The table on the right side of the figure is a summary of phenotype for the mutants lacking each cell-fusion gene. The severity of the alterations is represented with a gray circle (reduced /increased number or abnormal shape) and a black circle (structure not formed). The absence of a circle indicates that not phenotype has been described. PP, Protoperithecia; P, Perithecia; A, Ascospores. Phenotype data were obtained from (1).

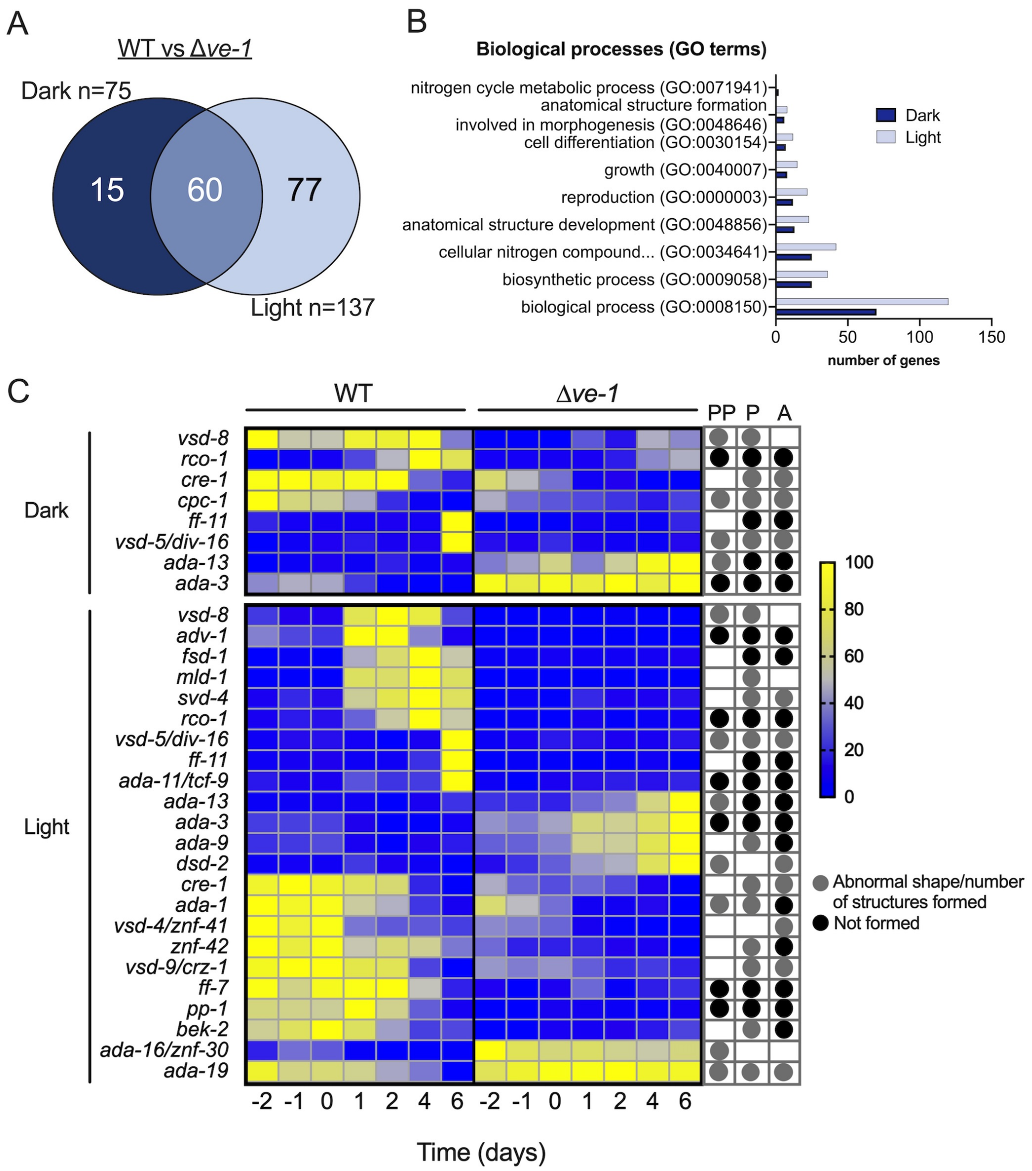

Fig. S10. Transcriptional profile of genes involved in transcriptional regulation. (A) Venn diagram showing the number of TFs displaying differential expression in the Δ*ve-1* mutant during sexual development in dark or light. Protoperithecia were fertilized at time 0. (B) GO term analysis showing enrichment of categories in each treatment. (C) Heat map representing normalized transcription levels (TMMs) of TFs genes with a mutant phenotype in sexual development (PP for protoperithecia; P for Perithecia, and A for ascospores). DEGs were identified after the analysis performed by magSigPro. Phenotype data were obtained from (1). The severity of the alterations is represented with a gray circle (reduced /increased number or abnormal shape) and a black circle (structure not formed). The absence of a circle indicates that not phenotype has been described.

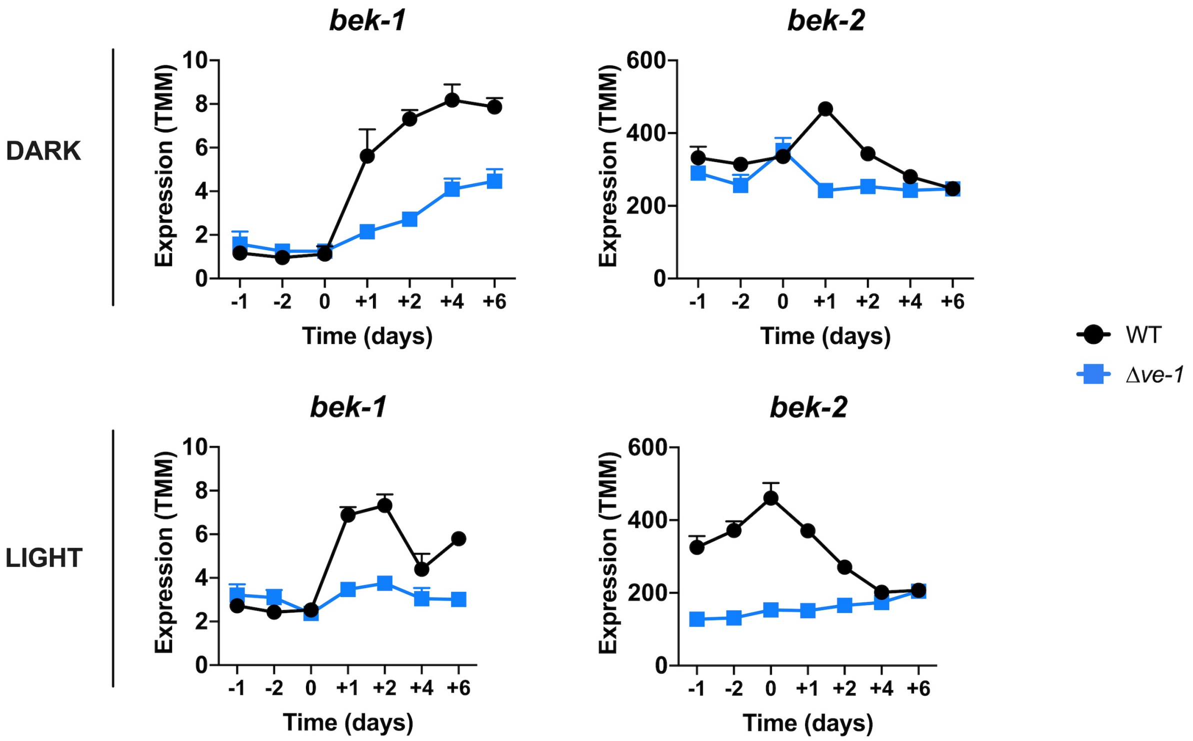

Fig. S11. Expression of *bek-1* and *bek-2* during sexual development. Expression (relative normalized transcription levels, TMM) of the transcription factor genes *bek-1* and *bek-2* in the wild type and the Δ*ve-1* mutant during sexual development in the dark or light. The plot shows the average and SE of three repeats from the RNAseq experiment. Protoperithecia were fertilized at time 0.

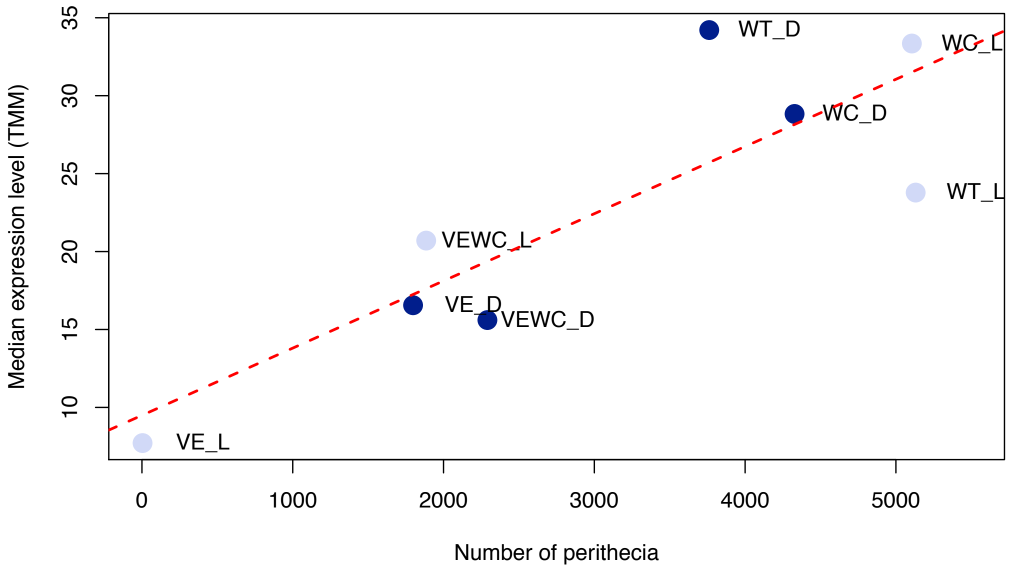

Fig S12. DEGs in the Δ*ve-1* Δ*wc-1* correlate with the number of perithecia. The decrease in the number of perithecia in the Δ*ve-1* mutant in light compared to dark must be due to changes in gene expression. We reasoned that we can shortlist those genes by comparing the Δ*ve-1* with the double mutant Δ*ve-1* Δ*wc-1* in light (i.e. a Δ*ve-1* mutant that is blind), because the double mutant showed similar number of perithecia in light than the single Δ*ve-1* mutant in the dark. The 272 genes identified were checked for genes that were differentially regulated in the Δ*ve-1* mutant in light during the time course experiment, providing a final list of 191 genes. The plot shows the correlation between the median expression of the genes in each genetic background and the number of perithecia in each light or dark condition. Light blue dots depict samples in light, dark blue dots depict samples in dark. The regression line is shown in red (r^2 = 0.85). Strains and conditions are: WT: wild type, VE: Δ*ve-1*, WC: Δ*wc-1*, VEWC: Δ*ve-1* Δ*wc-1*. L: light, D: dark.

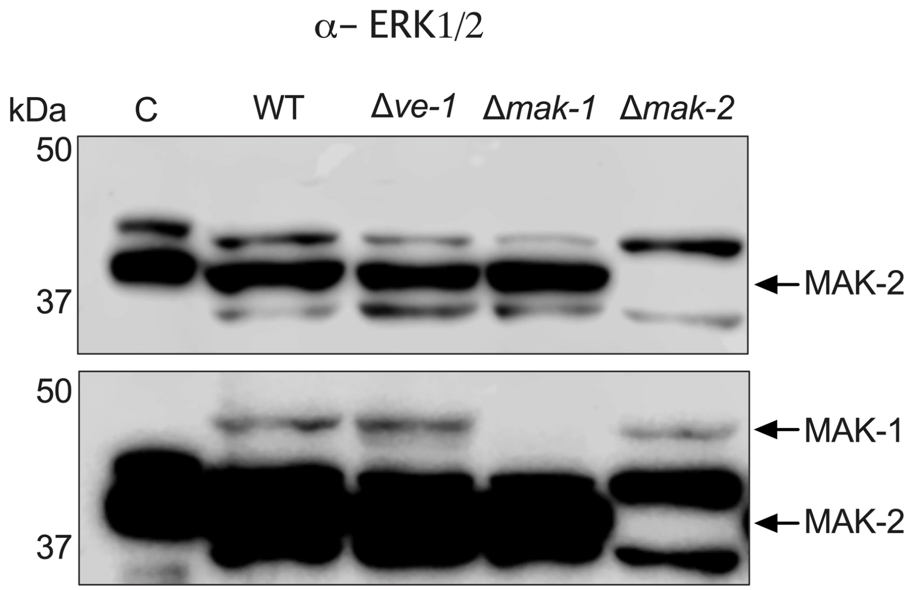

Fig S13. Accumulation of proteins for the components of the MAPK cascades MAK-1 and MAK-2. Protein samples from vegetative mycelia of wild type, Δ*ve-1*, Δ*mak-1* and Δ*mak-2* strains were separated by PAGE. Membranes were hybridized antibodies against MAP Kinase (ERK-1, ERK-2), and tubulin. 100 µg of proteins were loaded per lane. The lower pannel has been overexposed to observe MAK-1. We used as a positive control (C) a protein extract from Sorafenib-induced HepG2 cells (2).

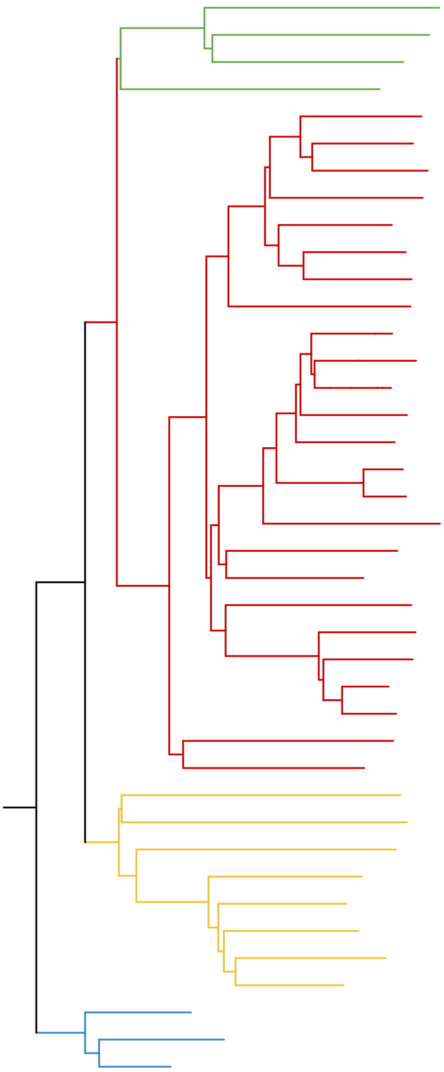

N. crassa homolog:	PR: *nrc-1*	PR: *mek-2*	PR: *mak-2*	CWI: *mak-1*	OS: *os-4*
Saccharomyces cerevisiae	2	1	2	3	2
Candida albicans	2	1	2	2	1
Pichia pastoris	2	1	2	2	1
Schizosaccharomyces pombe	2	1	1	1	2
Neurospora crassa	2	1	1	1	1
Myceliophthora thermophila	2	1	1	1	1
Podospora anserina	2	1	1	1	1
Magnaporthe grisea	2	1	1	2	1
Colletotrichum graminicola	2	1	1	1	1
Trichoderma reesei	2	2	1	1	1
Fusarium graminearum	2	1	1	1	1
Botrytis cinerea	2	2	1	1	1
Aspergillus fumigatus	2	1	1	1	1
Aspergillus nidulans	2	1	1	1	1
Aspergillus niger	2	1	1	1	1
Penicillium chrysogenum	2	1	1	1	1
Monascus ruber	2	1	1	1	1
Talaromyces marneffei	2	1	0	1	1
Talaromyces stipitatus	2	1	0	2	1
Histoplasma capsulatum	2	1	1	1	1
Cladonia grayi	2	1	1	1	1
Xylona heveae	2	1	1	1	1
Cladosporium fulvum	2	1	1	1	1
Stagonospora nodorum	2	1	1	1	1
Leptosphaeria maculans	2	1	1	1	1
Alternaria alternata	2	1	1	1	1
Cochliobolus heterostrophus	2	1	1	0	1
Arthrobotrys oligospora	2	1	1	1	1
Tuber melanosporum	2	1	1	1	1
Puccinia graminis	3	1	3	1	1
Ustilago maydis	2	1	2	1	1
Cryptococcus neoformans	2	1	2	2	1
Schizophyllum commune	3	2	2	2	1
Pleurotus ostreatus	2	1	3	2	1
Amanita muscaria	4	2	3	2	1
Coprinopsis cinerea	3	2	2	2	1
Laccaria bicolor	3	2	3	2	1
Phycomyces blakesleeanus	3	3	2	3	1
Rhizopus delemar	6	1	2	2	1
Mucor circinelloides	4	1	1	5	1

Fig S14. Distribution of homologs of *N. crassa* MAP kinase pathway genes regulated by VE-1 in the fungal kingdom. Homologs of components of the three MAP kinase pathways that are regulated by VE-1 in *N. crassa* (*nrc-1, mek-2* and *mak-2* in the PR, *mak-1* in the CWI and *os-4* in the OS pathway) were identified using Orthofinder, as previously reported (3). Orthogroups were identified using the *N. crassa* homologs, and confirmed by the presence of the corresponding *S. cerevisiae* homolog. The intensity of the green color is proportional to the number of homologs identified. The phylogenetic tree of species was also constructed using Orthofinder. Branches were colored according to the phylogenetic group: Ascomycetes yeasts (green), Ascomycetes filamentous fungi (red), Basidiomycota (yellow) and Zygomycota (blue).

Table S1. List of strains used in this study.

Strain	Genotype	Source
N. crassa		
**FGSC#2489**	4-OR23-1V *mat A*	FGSC
**FGSC#2490**	ORS-SL6a *mat a*	FGSC
**FGSC#11401**	*ve-1^{KO} mat A*	FGSC
**FGSC#11400**	*ve-1^{KO} mat a*	FGSC
**FGSC #13050**	*ve-2^{KO} mat a*	FGSC
**FGSC #13536**	*vos-1^{KO} mat A*	FGSC
**FGSC #22596**	*lae-1^{KO} mat a*	FGSC
**FGSC #22596**	*lae-1^{KO} mat a*	FGSC
**FGSC #11320**	*mak-1^{KO} mat A*	FGSC
**FGSC #11482**	*mak-2^{KO} mat A*	FGSC
**FGSC #11701**	*wc-1^{KO} mat a*	FGSC
**∆*ve-1* ∆*wc-1***	*ve-1^{KO} wc-1^{KO}*	(4)
**∆*wc-1 ve-1*FLAG**	*wc-1^{KO} ve-1^{FLAG}*	(4)
**SC-∆*ve-2***	*ve-2^{KO} mat A*	This study
**SC-∆*vos-1***	*vos-1^{KO} mat a*	This study
**SC-∆*lae-1***	*lae-1^{KO} mat A*	This study
**SC- ∆*ve-1*∆*ve-2***	*ve-1^{KO} ve-2^{KO}*	This study
**VE-2::3xFLAG**	*ve-2::10xGly3xFLAG*	(5)
**VE-1::3xFLAG; LAE-1::3xHA**	*ve-1::10xGly3xFLAG, lae-1::10xGly3xHA*	(5)
E. coli		
DH5α	*huA2 lac(del)U169 phoA glnV44 Φ80' lacZ(del)M15 gyrA96 recA1 relA1 endA1 thi-1 hsdR17*	(6)
ER2566	*F−λ−fhuA2 ompT lacZ::T7gene1 gal1 sulA11 Δ(mcrC-mrr)114::IS10(R9mcr-73::mini-Tn10-Tets) 2R(zgb-210::Tn10) (Tets) endA1*	NEW ENGLAND BioLabs

Table S2. List of primers used in this study.

Primers	Sequence 5'-3'	Use
**F-ve-1-Inteína TAG-sc**	GGTCATATGATGGGTGCTCAGGTTATCG	PCR
**R-ve-1-Inteína TAG-sc**	CGC TGCTCTTCCGCA ATACCCGCCAATATCTGC	PCR
**F-ve-2-Inteína TAG-sc**	GGTCATATGATGCAACCGTACACTTCAA	PCR
**R-ve-2-Inteína TAG-sc**	GGC TGCTCTTCCGCA ATAATCATCATCATCCTCATTA	PCR
**5'F mak-1Region1-6FAM**	CATCATTCGTCAGGAACCGACAAG	PCR
**3'R mak-1Region1**	TGGCGAATGTGTCTGTGTGGTGT	PCR
**5'F mak-2Region1-6FAM**	CAGTCTTGCTGCTGAGCCGG	PCR
**3'R mak-2Region1**	TTCGACGTGTCCCGAAAAGTTGC	PCR
**5'F mek-2Region1-6FAM**	GCTGCCAAGATACGTTAAGACAAAGG	PCR
**3'R mek-2Region1**	GATGTCTGTTTGTTTAAGCGTAGTCG	PCR
**5'F os-4 Region2-6FAM**	GACCGGGACTGGCAGTGAC	PCR
**3'R os-4 Region2**	TGTCGCCGACAAGTCAAAGG	PCR

Data Set S1. RNAseq of the wild type and the Δ*ve-1* mutant during sexual development. CPM (Counts per kilobase per million) values for all probes.

Data Set S2. Characterization of the regulation of transcription during sexual development of *N. crassa* wild-type and Δ*ve-1* strains.

Data Set S3. Genes regulated by VE-1 during sexual development

Data Set S4. Genes regulated by VE-1 required for sexual development.

Data Set S5. Genes regulated in the Δ*ve-1*, Δ*wc-1*, and Δ*ve-1* Δ*wc-1* mutant strains in sexual development (time -1).

Data Set S6. Coordinated regulation of genes involved in fruiting body formation by VE-1 protein and the MAP kinases MAK-1 and MAK-2

References

1. Carrillo AJ, Cabrera IE, Spasojevic MJ, Schacht P, Stajich JE, Borkovich KA. 2020. Clustering analysis of large-scale phenotypic data in the model filamentous fungus *Neurospora crassa*. BMC Genomics 21:755.
2. Contreras L, Rodríguez-Gil A, Muntane J, de la Cruz J. 2022. Broad transcriptomic impact of Sorafenib and its relation to the antitumoral properties in liver cancer cells. Cancers (Basel) 14.
3. Ojeda-López M, Chen W, Eagle CE, Gutiérrez G, Jia WL, Swilaiman SS, Huang Z, Park HS, Yu JH, Cánovas D, Dyer PS. 2018. Evolution of asexual and sexual reproduction in the aspergilli. Stud Mycol 91:37-59.
4. Gil-Sánchez MDM, Cea-Sánchez S, Luque EM, Cánovas D, Corrochano LM. 2022. Light regulates the degradation of the regulatory protein VE-1 in the fungus *Neurospora crassa*. BMC Biol 20:149.
5. Sarikaya-Bayram Ö, Dettmann A, Karahoda B, Moloney NM, Ormsby T, McGowan J, Cea-Sánchez S, Miralles-Durán A, Brancini GTP, Luque EM, Fitzpatrick DA, Cánovas D, Corrochano LM, Doyle S, Selker EU, Seiler S, Bayram Ö. 2019. Control of development, secondary metabolism and light-dependent carotenoid biosynthesis by the velvet complex of *Neurospora crassa*. Genetics 212:691-710.
6. Taylor RG, Walker DC, McInnes RR. 1993. *E. coli* host strains significantly affect the quality of small scale plasmid DNA preparations used for sequencing. Nucleic Acids Res 21:1677-8.
